# Biosensor Technologies for Avian Influenza Detection: A New Frontier in Rapid Diagnostics for HPAI

**DOI:** 10.3390/bios16020118

**Published:** 2026-02-12

**Authors:** Jacquline Risalvato, Alaa H. Sewid, Durina Z. Dalrymple, Shigetoshi Eda, J. Jayne Wu, Richard W. Gerhold

**Affiliations:** 1 Biomedical and Diagnostic Sciences, College of Veterinary Medicine, The University of Tennessee, Knoxville, TN 37996, USA; rgerhold@utk.edu; 2 School of Natural Resources, The University of Tennessee Institute of Agriculture, Knoxville, TN 37996, USA; asewid@utk.edu (A.H.S.); seda@utk.edu (S.E.); 3 Department of Microbiology, The University of Tennessee, Knoxville, TN 37996, USA; 4 Department of Electrical Engineering and Computer Science, The University of Tennessee, Knoxville, TN 37996, USA; jwu10@tennessee.edu

**Keywords:** avian influenza diagnostics, biosensors, DIVA (Differentiating Infected from Vaccinated Animals), HPAI (Highly Pathogenic Avian Influenza), point-of-care diagnostics, One Health surveillance

## Abstract

Avian influenza (AI), particularly highly pathogenic avian influenza (HPAI), represents a serious and growing threat to global poultry production, international trade, and human health security. Control of AI is complicated by the high evolutionary rate of influenza A viruses, which drives antigenic diversity and ongoing emergence of novel strains. Effective surveillance and disease management therefore depend on timely and accurate diagnostics. While conventional methods—including virus isolation, reverse transcription-quantitative polymerase chain reaction (RT-qPCR), and enzyme-linked immunosorbent assays (ELISAs)—remain effective and widely used, they are limited by long turnaround times, the need for specialized equipment, and reliance on highly trained personnel. In addition, strict state and federal regulatory requirements restrict testing to a limited number of authorized laboratories. Although these regulations are essential for maintaining diagnostic accuracy and quality assurance, they place substantial strain on laboratory capacity during outbreaks and delay actionable results. The need for rapid, on-site decision making has driven interest in alternative diagnostic approaches, including biosensor technologies. A major limitation of current diagnostic strategies is the lack of robust DIVA (Differentiating Infected from Vaccinated Animals) capability. In countries such as the United States, where poultry vaccination against AI is not routinely practiced, the absence of DIVA-compatible diagnostics has hindered adoption of vaccination as a disease management tool, as seropositive birds and products face significant trade restrictions. Biosensor platforms capable of enabling DIVA strategies offer a potential pathway to support vaccination while preserving surveillance integrity. This review examines the current landscape of AI and HPAI diagnostics, emphasizing the limitations of traditional approaches and the opportunities presented by biosensor platforms. We evaluate electrochemical, optical, piezoelectric, and nucleic-acid-based biosensors, with particular attention to biorecognition strategies, performance metrics, field deployability, and applications supporting subtype discrimination, DIVA implementation, and One Health surveillance.

## 1. Introduction

### 1.1. Background on Avian Influenza Viruses (AIVs)

Influenza A viruses (IAVs) are veterinary and human health pathogens with a worldwide presence and centuries if not millennia of history. All influenza viruses belong to the viral family *Orthomyxoviridae*, a hallmark of which is viruses possessing segmented negative-sense RNA genomes [1]. The genera of Orthomyxoviruses include influenza types A, B, C, and D, Isaviruses, and Thogotoviruses. Influenza B and C are viruses of primarily human importance and rarely infect other species [2]. Influenza D is an influenza virus primarily of cattle with occasional spillover into other species, primarily swine [3]. Influenza A viruses, on the other hand, infect a plethora of different avian and mammalian species worldwide, including but not limited to humans, birds, marine mammals, such as whales and seals, felines, canines, swine, equids, bovids, and more [1,2].

The structure of IAVs rests on its segmented genome—all influenza viruses have eight gene segments encoding at least ten different viral proteins. The two surface proteins that are responsible not only for the virus binding to host cells and cell egress but also viral subtype naming are hemagglutinin (HA) and neuraminidase (NA). Other proteins of note in influenza virus are the membrane ion channel proteins (M2) and internal proteins, such as the nucleoprotein (NP), the matrix protein (M1), the polymerase complex containing polymerase basic protein 1 (PB1) and 2 (PB2), and the polymerase acidic protein (PA) [1,4]. Nonstructural proteins of influenza viruses are nonstructural proteins 1 (NS1) and 2 (NS2), along with nuclear export protein (NEP); the nonstructural proteins of influenza viruses are often critical in host–cell interactions during viral replication and virion maturation [5].

Avian influenza viruses (AIVs), originally called “Fowl plague” when first recognized in the 1870s in Europe, had spread rapidly in Europe and Asia with reports in North and South America before the Great Depression [2,6]. The term “avian influenza” refers to any IAV that primarily affects birds but is not limited to affecting other species, such as mammals, including humans [7,8]. The primary reservoir of AIVs is wild aquatic birds, and they are considered enzootic in many bird populations globally [9]. AIVs are of the HA subtypes 1–16 and NA subtypes 1–9 in a variety of combinations and preferences. For example, the H1–13 subtypes of AIVS are found often in poultry, whereas sporadic cases of H2, H4, H5, H7, and H9 affect pigs with endemic circulation of H1 and H3 subtypes [2]. Canine influenza development was due to two separate AIV spillover events: H3N8 from equine species, which originated from wild birds and poultry, and H3N2, which originated from a sporadic event in poultry [10]. Aiding and abetting AIV and IAV epidemics and pandemics across species is the virus’ unique ability to undergo genetic rearrangement, the process through which virions can “mix and match” their gene segments to undergo rapid antigenic shift. Viral reassortment is also not a process lost on IAVs and assists along with natural RNA virus mutability with antigenic drift events [2,11]. The species availability, high genetic mutability, ability of the virus to reassort and rearrange genetic segments, and transmissibility are all critical compounding factors of influenza epidemic and pandemic development.

**Figure 1:**
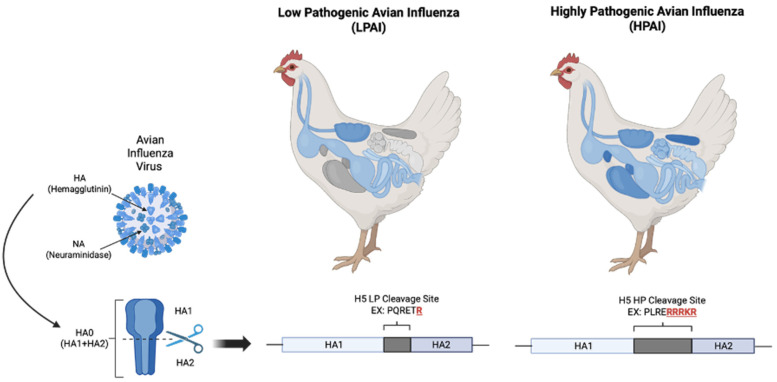
Low versus highly pathogenic avian influenza hemagglutination changes and pathologic outcomes. For AIV to be active in the host, its external hemagglutinin (HA) protein needs to be proteolytically cleaved from HA0 to HA1 and H2. Low pathogenic avian influenza (LPAI) is restricted to the upper respiratory tract and gastrointestinal tract of poultry due to the cleavage site (gray) of the hemagglutinin protein (HA0) being limited body sites where it can be recognized by host proteases for cleavage (organ systems in blue). Highly pathogenic avian influenza (HPAI), on the other hand, has the addition of multiple basic amino acids to the cleavage site of HA1–2 (red text), allowing for ubiquitous cleavage by host proteases throughout the body. Created in BioRender. Risalvato, J. (2025) https://BioRender.com/sra42ks.

When discussing AIVs, it is important to understand the differences between highly pathogenic (HP) and lowly pathogenic (LP) AIs. LPAI causes limited disease morbidity and mortality in birds, and its viral pathogenicity is restricted to the gastrointestinal and respiratory tracts. HPAI, on the other hand, acquires an additional polybasic cleavage site in its HA protein that allows for ubiquitous cleavage of sialic acids. Normally, HA cleaves α-2,3-sialic acids that are restricted to epithelial cells of the respiratory and gastrointestinal tract. However, the addition of this cleavage site allows for cleavage of α-2,6-sialic acids as well, which are found in all epithelial cells in the avian host as well as the lower respiratory tracts of humans [12,13]. The expansion of the virus’ ability to infect a smorgasbord of host cells throughout the host is the defining factor for these viral strains to be classified as highly pathogenic, which is showcased in Figure 1. When compared to LPAI, where up to 30% mortality could be expected in a domestic flock, HPAI mortality rates can be as high as 100% within 24 to 48 h of the onset of clinical signs in a flock. HPAI infections in humans, of which over 500 cases have been confirmed since 2003 in 15 countries, have a case fatality rate of nearly 60% [14]. HPAI subtypes appear to be restricted to only H5 and H7, but not all H5 and H7 subtypes are highly pathogenic—there are LPAI strains of both subtypes, as well. The only way to truly identify a HPAI subtype is to identify the presence of a polybasic cleavage site in the HA. All HPAI virus strains are reportable both in the US and internationally, and any event of AI in a domestic flock or mammalian species is considered reportable to the USDA.

Taken together, influenza A viruses’ genetic versatility, wide host range, and variable pathogenicity—particularly those of avian subtypes—highlight their ongoing danger to both human and animal health and emphasize the significance of prompt identification and close observation, particularly when dealing with highly pathogenic strains.

### 1.2. Global Impact of Highly Pathogenic Avian Influenza (HPAI)

Global poultry production, food security, and public health are all threatened by the emergence and spread of HPAI. Since 2005, over 500 million poultry animals have succumbed to disease or been culled worldwide due to HPAI outbreaks, with dire economic and social repercussions [15]. In addition to direct losses from HPAI’s high mortality rates in poultry and extensive depopulation efforts to control disease spread, these effects also involve higher biosecurity expenditures and changes in consumer behavior in response to media-reported perspectives [16,17]. Additionally, growing concerns and evidence regarding the transmissibility and broad host range of HPAI bring about increased concern for other food source production markets.

The 2022 HPAI outbreak in the United States has been considered one of the most important and devasting animal health incidents in national history—over 58 million lives of poultry animals have been claimed due to disease and culling practices to maintain the outbreak [18]. Poultry supply chains were greatly impacted by this disruption, particularly the egg industry, where a shortage of mature laying hens and eggs resulted in record-high egg prices for consumers—often above USD 6/dozen on average nationwide. The decline of USD 2.6 billion in economic output, losses of almost USD 1 billion in value added, and the loss of over 15,000 jobs and means of subsistence were among just some of the vast economic consequences of this ongoing outbreak. Additionally, state and federal tax revenues decreased by approximately USD 136 million and USD 248 million, respectively. Consumer markets are also heavily impacted—in the United States, a recent analysis estimated a USD 1.41 billion loss in consumer surplus due to elevated egg prices during the 2022 HPAI outbreak [18,19]. These price spikes disproportionately affect lower-income households in search of a typically affordable and widely available protein option and can lead to long-term changes in consumption patterns and market demand. Europe experienced a similar crisis with this outbreak, with over 48 million birds culled across 37 countries between 2021 and 2022 [18], reflecting the transboundary nature of the disease and the vulnerability of even highly regulated and markets.

In addition to the financial strain induced by major food supply losses, HPAI has significantly impacted public health around the world. Due to their historically high human case fatality rates, ability to genetically reassort with influenza strains that infect both other animals and humans, and the potential for zoonotic transmission, HPAI viruses pose serious threats to public health. Despite being considered a relatively rare event, human HPAI viral infections are frequently quite serious, with mortality rates varying between 50 and 60%, largely due to their ability to replicate deep in the lower respiratory tract, where α-2,6-linked sialic acids are found [13,20]. Luckily, to date, there has been no documented case of human-to-human HPAI transmission.

The primary concern in public health is the AI’s potential to adapt for efficient human-to-human transmission. Although sustained transmission has not yet occurred, the virus’s segmented genome enables reassortment with human influenza viruses in co-infected hosts, increasing the risk of generating a pandemic strain—especially in the face of HPAI spread [21,22,23]. Mammals, such as swine, which can be infected with both avian and human influenza viruses, act as “mixing vessels” for such reassortment events—as seen in the US 2009 pandemic of swine influenza H1N1 [13,14].

The recent emergence of HPAI H5N1 infections in dairy cattle in the United States underscores the virus’s expanding host range and the associated zoonotic risk. Viral RNA has been detected in unpasteurized milk and nasal secretions of infected cattle, suggesting potential routes for human exposure [24,25]. While pasteurized dairy products are considered safe, the detection of the virus in raw milk and the confirmed human case linked to cattle exposure in 2024 highlight the importance of occupational biosecurity and surveillance in agricultural settings [26]. Experimental research has revealed that certain mammalian species, such as cats and ferrets, are extremely vulnerable to HPAI infection, with respiratory transmission in close contact situations having been proven to occur [22,27]. Concerns regarding possible contamination of domestic animals, household pets, and wildlife have been increasingly raised, as these occurrences could support larger networks of transmission.

The necessity of concerted international actions is becoming more widely acknowledged in light of HPAI’s worldwide economic and public health impact. A ten-year global strategy has recently been launched in a joint effort between the Food and Agriculture Organization (FAO) and the World Organization for Animal Health (WOAH) to prevent, monitor, and respond to HPAI threats [28]. Investments in vaccine development, transboundary and cross-border effective communication strategies, early detection systems, and strict biosecurity procedures are essential components of this global approach. Given these risks, HPAI viruses continue to pose a real zoonotic threat in addition to being a persistent threat to the world’s poultry production industries. Mitigating the threat of zoonotic and pandemic influenzas requires effective monitoring of viral evolution, rapid diagnostic tools, vaccination strategies, and One Health approaches to biosecurity.

### 1.3. Limitations of Conventional Diagnostics

The two main principles of current AI diagnostics are (1) identifying the virus’ acute presence through viral antigen or protein or (2) identifying the virus’ past presence by examining the host’s immune responses through serology. While agar gel immunodiffusion (AGID), ELISAs, hemagglutination inhibition assays (HAI or HI), and neuraminidase inhibition assays (NAI or NI) are used to detect AI immune responses, viral isolation, real-time RT-qPCR, and hemagglutination assays are used to detect AI antigenic presence. Only laboratories that are a part of the National Animal Health Laboratory Network, or NAHLN, which is subject to stringent international standard accreditations and frequent audits, are permitted to perform any one of these assays, which are strictly regulated by yearly proficiency training and certification requirements.

While there are various validated diagnostic methods for HPAI, there are still several obstacles that prevent prompt detection and action in outbreak responses. The regulatory complexity surrounding the use of diagnostic tools, especially in field settings, is a major obstacle. Under stringent biosafety and quality control regulations, standard diagnostics like RT-qPCR are centralized in government or regional laboratories under national veterinary authorities [29]. While these measures preserve quality assurance and diagnostic integrity, as well as biosafety for human personnel, these procedures slow down the adoption of more recent, potentially field-deployable technologies due to drawn-out approval procedures and a cautionary approach to validation outside of conventional laboratory settings [30,31].

Another obstacle to consider is cost, particularly for extensive surveillance programs and in environments with limited resources. Traditionally, state and federal governments subsidize testing costs for producers, but due to strict regulation of the test, cost is still a concern, and, typically, surveillance is not extensively pursued beyond cases where there is likely to be a rampant AI outbreak occurring. Despite being the gold standard, molecular assays like RT-qPCR and even virus isolation are too costly for routine screening due to their dependence on complex equipment, high-purity reagents, and hefty cold-chain logistics [32]. Antigen-based rapid diagnostic tests (RDTs), on the other hand, are more economical but less reliable in identifying low-viral-load or emerging HPAI strains due to their poor subtype differentiation and lower sensitivity [29,33].

Furthermore, the scalability of existing diagnostics is further constrained by their technical complexity and the requirement for qualified, highly trained technical staff. Trained laboratory personnel skilled in sample handling, viral nucleic acid extraction, and result interpretation are necessary for proper execution of molecular assays, as serological assay interpretation success often hinges on increased experience and frequent proficiency in testing until a technician is cleared. Early outbreak detection and reporting are hampered in many rural or resource-constrained areas by a lack of specialized training, and these rural areas are often demographically where poultry production facilities reside [32]. Additionally, the turnaround time for confirmatory diagnostics, which can exceed 24–48 h when sample batching and transportation are considered, can cause containment measures to be delayed that would be critical to minimizing viral transmission windows.

Lastly, detection and assay design are complicated by the rapid, ever changing natural of AIVs. Assays designed for previously circulating strains may not be sufficient at detecting novel genotypes due to high mutation rates, antigenic drift, and frequent reassortment events [34]. Because of this ingoing viral evolution and host species transmission dynamics, molecular primers and antibodies used in diagnostics must be updated on a frequent basis—requiring numerous resources and lags in the face of real-time viral dynamics [35,36]. As a result, current HPAI diagnostics are limited by biological uncertainties inherent in influenza virus evolution in addition to logistical and technological obstacles.

### 1.4. Scope and Objectives of This Review

The objective of this review is to critically evaluate the current state of AI diagnostic technologies, emphasizing the detection of HPAI and the growing contribution of biosensor platforms to the transformation of field-level surveillance and response. While traditional diagnostic techniques and next-generation sequencing continue to be the gold standard for HPAI confirmation, they are severely limited in their ability to adapt to the changing genetic landscape of the virus, their cost-effectiveness, and their flexibility regarding technical staff and regulations. These restrictions pose a serious obstacle to prompt containment and responses to outbreaks, especially in decentralized or resource-constrained agricultural environments.

To address these gaps, this review concentrates on recent innovations in biosensor technologies that promise to deliver rapid, sensitive, and portable diagnostics suitable for on-site application. We explore a wide array of biosensing modalities, including electrochemical, optical, nucleic-acid-based, and CRISPR-enabled systems, evaluating their mechanisms of biorecognition, detection performance, and practical deployment potential in farm and field contexts. Special attention is paid to the integration of nanomaterials, aptamers, and microfluidics to enhance sensitivity and specificity, especially for detecting viral HA and NA subtypes critical for subtype identification and pathogenicity assessment.

Examining the biosensor technologies supporting DIVA strategies—a crucial but underdeveloped area in HPAI diagnostics—is one of the main goals of this review. Diagnostic platforms that can distinguish between infection-induced and vaccine-induced immune responses are desperately needed as vaccination strategies become the more practical and desirable option in preventing transmission in the face of rampant outbreaks, especially when considering zoonotic risks and trade effects. A promising way to enable safe vaccination programs without sacrificing the integrity of epidemiological surveillance is using biosensors that can identify strain-specific antibodies, conserved viral antigens, distinct molecular markers of infection, or various mutations linked to HPAI emergence.

Ultimately, this review seeks to highlight how biosensor-based diagnostics could serve as a disruptive force in HPAI monitoring—enhancing early warning systems, enabling DIVA-compatible surveillance, and supporting One Health goals by bridging the gap between laboratory-grade accuracy and field-ready practicality.

## 2. Current Diagnostic Approaches for Avian Influenza

Molecular and serological techniques dominate the diagnostic field for AI confirmation. Table 1 provides an overview of the primary diagnostic methodologies described in this section, including their specialty uses and drawbacks.

### 2.1. Molecular Techniques

Molecular-based assays, such as polymerase chain reaction (PCR), are often used for the detection of minute amounts of viral genomic material in a variety of sample matrices. PCR can either be conventional, where primers are used to amplify a given genetic sequence and that product undergoes gel electrophoresis for visualization by the human eye, or real-time quantitative techniques where, along with the primers, a fluorescent probe is used to quantify the copy number of nucleic acid product. RT-qPCR is the molecular method of choice for most diagnosticians for identification of IAV nucleic acids due to it being quicker and more sensitive than conventional RT-PCR methods [31]. Currently, RT-qPCR primarily identifies the presence of IAV matrix (M) gene in a sample, and that ample will then undergo either more molecular diagnostics (HA and NA subtyping) and/or next-generation sequencing (NGS) at the National Animal Health Laboratory Network (NAHLN) headquarters, the National Veterinary Services Laboratory (NVSL) in Ames, Iowa. Multiplexing can be used by authorized labs to target multiple targets simultaneously but is not often utilized in diagnostic settings [29,37].

Overall, RT-qPCR molecular methods provide timely and efficient sensitive and specific diagnostic options for laboratories for the identification of viral presence in simple and complex samples. However, the procedure requires proficiency training by adept personnel not only in running the test but in extracting fragile RNA from biological or environmental samples, higher turnaround times when confirmation of test results is required, and highly specialized equipment and reagents not available to all laboratories or locations, and it can be cost-prohibitive when attempting surveillance with multiple samples.

Loop-mediated isothermal amplification (LAMP) provide another molecular alternative on the scene in AI diagnostics. The method involves amplifying a target sequence under isothermal conditions using a DNA polymerase and four to six primers, with two of these primers being “looping” primers and two being “stripping” primers. These primers are designed to, when bound to their target nucleic acid, form loop structures to enable polymerase-based amplification. The loop structures formed are not only specific for identification by Bst polymerase but also allow for multiple sites of initiation to increase amplification and product yields [29]. Arguably the best benefit of the LAMP molecular assay design is that it can be performed at ambient temperature, removing the requirement for specialized equipment and temperature-sensitive reagents, making it affordable and field deployable [38]. The final product can be identified through gel electrophoresis or the use of fluorescent probes for more sensitive quantification, which can be detected by either a laboratory-based fluorometer or by the naked eye via a color change in the sample tube. LAMP assays have been designed for human H1 and H3 and avian H5, H7, and H9, and they have been multiplexed in their research applications [39,40].

Compared to widely used molecular methods, LAMP provides the benefit of foregoing expensive diagnostic equipment in favor of field deployability. However, there are some limitations to LAMP, one being that designing four to six primers for one assay can be challenging and requires specialized research and technical support for development. Additionally, LAMP primers require the selection of six to eight unique regions on the target gene, adding to the challenges of assay design [41]. Lastly, the use of multiple primers increases the odds of primer–primer interactions, which can lead to false positive results and decrease assay specificity.

A more recent method used by highly specialized approved laboratories is sequence analysis. Advancements in automated sequencing technology have allowed for the determination of the presence of the HA proteolytic cleavage site, the determining factor for LPAI vs. HPAI virus, achievable in under 24 h. These sequencing endeavors also assist in molecular epidemiology and tracking viral evolution patterns. Because AI has a segmented genome, all eight segments need to be sequenced for complete analysis of changes in the virus that can affect pathogenicity, transmissibility, and species preference [42,43]. However, the cost of next-generation sequencing and the time to both produce and analyze the data by trained epidemiologists and personnel limit the turnover of isolates and their information that can be evaluated by reference and research laboratories.

**Table 1:** Evaluation of the traditional diagnostic assay, its target, relative sensitivity, specificity, cost, time to result, personnel requirements, best use cases, and notes. Adapted from Avian Influenza Diagnostics and Surveillance Methods, Avian Influenza 2008 [43].

Diagnostic Assay	Target	Relative Sensitivity	Relative Specificity	Estimated Cost per Sample	Required Expertise	Time to Result	Use Case	Notes
Virus isolation	Viable virus	High	Moderate	$$$	High	1 to 3 weeks	Confirmatory testing, strain isolation, research	Gold standard; requires biosafety level 3 (BSL-3) lab
Antigen detection	AIV protein	Low	High	$$	Moderate	15 min	Rapid screening at point of care	May yield false negatives in low-viral-load samples
Real-time RT-PCR	AIV RNA (M protein)	High	High	$$	Moderate	~3 h	Detection and subtype identification	Standard for surveillance; multiplexing possible
Agar gel immunodiffusion (AGID)	Antibody to IAV nucleoprotein and matrix protein	Moderate	Moderate	$$	High	48 h	Serological surveillance post-infection	Less sensitive than ELISA; requires intact serum antibody
ELISA	Antibody to IAV	Moderate	Moderate	$	Moderate	~2 h	Large-scale seroprevalence studies and flock surveillance	Can distinguish vaccinated vs. infected animals (DIVA-compatible formats) depending on vaccine design
Hemagglutination (HA) inhibition (HAI)	Identification of IAV HA subtype and subtype-specific antibodies	High	Moderate	$$	High	~2 h	HA subtyping, immune status evaluation (titer instead of positive vs. negative result)	Requires standardized RBCs and reference antigens; may require BSL-3
Neuraminidase (NA) inhibition (NAI)	Identification of IAV NA subtype and subtype-specific antibodies	High	Moderate	$$	High	~3 h	NA subtyping, strain differentiation	Used alongside HAI for full subtype identification
Loop-mediated isothermal amplification (LAMP)	AIV RNA	Moderate to high	High	$	Low to moderate	~30–60 min	Field diagnostics, resource-limited settings	Isothermal amplification; does not require a thermocycler
Next-generation sequencing (NGS)	Whole AIV genome	Very high	Very high	$$$$	Very high	Days	Whole-genome surveillance, variant detection	Requires bioinformatics infrastructure; current requirement for HPAI vs. LPAI differentiation
Microarray/DNA chip	Multiple AIV gene targets	High	High	$$$	High	~6 h	Simultaneous detection and subtyping of multiple strains	Less commonly used in field settings

Conclusively, molecular assays provide high sensitivity and specificity in the diagnosis of the acute presence of AI even with complex sample matrices, making them highly desirable as a testing modality for influenza. However, the requirements of highly trained personnel for assay implementation and design, cost-prohibitive equipment, and restricted use spaces, and the lack of access to non-laboratory areas where outbreaks often occur, are an obstacle to molecular diagnostics in AI outbreak responses.

### 2.2. Immunological Assays

Current immunological assays for AI measure the antibody levels of adaptive immune responses in the host. The four most common immunological methods utilized for AI detection are AGID, ELISA, and HA inhibition and NA inhibition assays (HIA and NIA, respectively).

**Figure 2:**
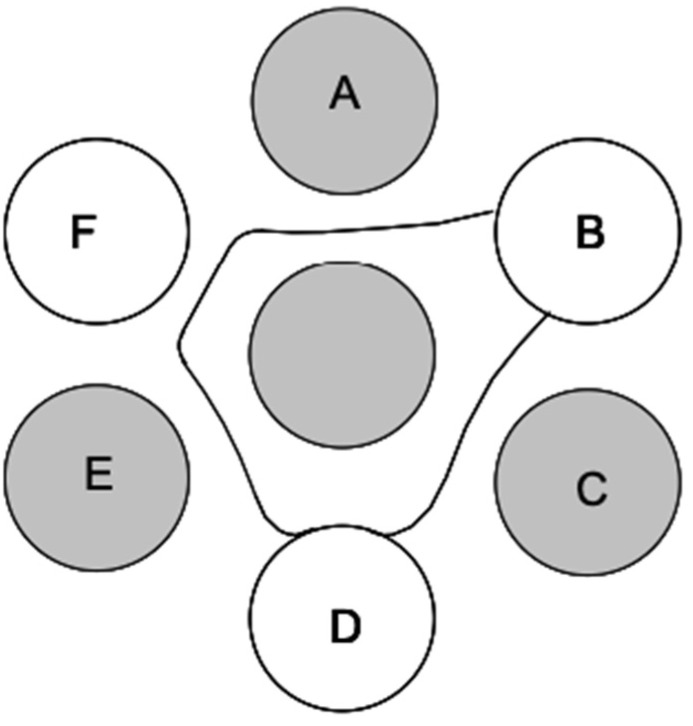
An agar immunodiffusion test pattern for AI-AGID. The reference antigen for IAV is in the central well (gray). Wells A, C, and E (gray) have positive reference serum—as such, there is a precipitation line between these wells and the central antigen well. The wells in white (B, D, and F) have various test sera. Well B contains a serum sample that is negative for IAV antibodies, well D contains serum that is weakly positive for IAV antibodies, and well F contains serum that is positive for IAV antibodies. This figure was obtained from the USDA Agar Gel Immunodiffusion Test to Detect Antibodies to Type A Influenza Virus NVSL-SOP-0045 protocol [46].

AGID operates on the principle of visualizing an immunoprecipitation reaction of AI virus antibody and antigen after diffusion towards one another in an agar gel matrix. An agar gel is poured with a seven-well pattern imprinted. The central well holes in the antigen, typically to either IAV NP or M protein, while the six surrounding wells alternate between test samples (up to three serum samples) and three known antiserum samples as positive controls. An AGID for AI can be visualized in Figure 2. AGID is widely used in diagnostic settings for the detection of antibody with a reference antigen but can also be used in IAV detection to confirm AI in allantoic fluid from embryonated eggs and virus isolation samples [44]. The AGID test is still routinely used in diagnostic facilities due to its inexpensive setup and relatively simple procedures, as it does not require unusual supplies or expensive equipment. But, preparation of the reagents with regulated quality assurance is expensive and time consuming and only available through federally approved reference laboratories. As such, these reagents do sometimes experience backorders and supply chain delays. Furthermore, the skill required to interpret test results requires regular proficiency testing and training that is also regulated by federal agencies. AGID results have a moderate turnaround, with results being read as early as 24 h but as late as 48 h in situations with weak positive results [45,46].

ELISA for AI can be done through a variety of commercial kits, and some of these kits offer type-specific detection of AI viral antibodies in matrices from serum, plasma, albumin, and egg yolk from chickens. ELISA provides higher sensitivity than AGID tests as it is not measured by the naked eye, but it could provide false positive results due to poorer specificity [47]. Despite this risk, ELISA can be performed much faster than AGID and is more applicable to high-throughput testing, which is perceived well by producers with large-scale farms and facilities that not only have tens of thousands of birds in their flock but may also be subjected to weekly screenings for biosecurity and regulatory requirements [43]. Most commercially available AI-ELISAs, though, are specific to poultry animals, such as chickens and turkeys, and do not provide accurate assessments from sera of other birds, such as ducks, geese, or waterfowl.

The HI assay is a serological test that can be used for confirmation of subtype-specific AI viruses to further characterize AI isolates through subtype identification or to identify subtype-specific antibodies and quantify their titers to AIV in either serum, plasma, or egg yolk. The assay operates by using a 96-well plate. An equivalent volume of erythrocytes (species-dependent based on the HA subtype being tested) is aliquoted across the wells of a singular row. Then, an equivalent volume of IAV pre-mixed with titrated serum sample is added to the erythrocyte-containing wells along the row. Should adequate antibodies be present in the sample, the antibodies should bind to the HA of interest and prevent hemagglutination of the erythrocytes by the virus. Steric inhibition, unfortunately, can lead to false positive results when there is a homologous NA present, but this problem can be overcome by using antisera prepared through DNA vaccines that were prepared using only the HA gene [48,49]. There may also be cross-reaction between certain HA subtypes, clouding interpretation. Some issues with specificity are that the matrix being tested must be confirmed clear of the presence of any other virus that can hemagglutinate, such as avian paramyxovirus type-1 (AMPV-1 or Newcastle Disease Virus) [43]. These factors make HI assay specificity dependent on the quality of the antibody panel and the subtype being tested. Additionally, HI is often considered more sensitive to AGID and will detect the presence of AI antibody for a longer period of post-exposure than AGID. Further, HI provides the ability to quantify antibody levels via titration. HI, while used primarily for poultry species, is not as species-specific as AGID and can be used across other species of birds.

NI works similarly to the principles of HI, but the assay is used to detect the NA subtype of IAVs via subtype-specific antibodies or antigen. As such, it is most frequently used for this method specifically. Rather than inhibiting the enzymatic activity of HA, subtype-specific antibodies are used to inhibit the enzymatic activity of the NA. Rather than clear-V bottom plates that are often used in HI assays, NI assays require white-colored plates to interpret color changes as, rather than looking for erythrocyte agglutination, the presence of a colorimetric reaction is observed if NA activity is not blocked by the presence of a subtype-specific antibody [43]. The sensitivity and specificity of NI are very similar to those of HI. However, NI is often more complicated than HI and takes a slightly longer period of time to perform. It is also more expensive than HI, as the substrate used in this test is expensive.

### 2.3. Virus Isolation and Culture

The gold reference standard for AI, as with most viral agents, is viral isolation (VI). It is the isolation of a virus from a sample in culture that confirms the presence of the virus and allows for further characterization. VI is typically done either in cellular culture or in cell lines, such as Madin–Darby canine kidney cells (MDCK). For LPAI viruses, trypsin must be added to cell culture media to facilitate HA protein cleavage to assist in successful replication in these limited outside-of-the-host systems. More often than not, passage in embryonated chicken eggs (at 9 to 11 days of incubation) is the preferred method for VI of AIVs, as it is considered to be the most sensitive system for isolation of suspected poultry-adapted AIVs and can be used with a variety of sample types, such as tissue homogenates from necropsies, cloacal, tracheal, and oropharyngeal swabs, and even environmental samples. Serial passage up to three times, though, may be required to achieve optimal sensitivity, increasing the time needed to complete the test [43,49,50].

VI of AIV is highly sensitive and can even detect active infection several weeks postexposure from residual viral shedding in the host and its tissues or the environment [45]. It is also not subject to species restrictions [51]. However, it is not a highly specific or selective assay, as other agents from a sample may readily grow in chicken eggs or cell cultures, such as AMPV-1. Additional tests on egg fluid or cell cultures are often required to confirm the presence and purity of AIV in the sample, such as HA or HI assays for subtyping or even molecular methods [43].

Additionally, VI is an expensive and time-consuming process due to the procurement of resources and the requirement of egg incubation. Furthermore, infectious viruses are amplified to a high level during the VI process, which can increase the potential for cross-contamination and biosecurity risks. VI is usually performed in laboratories with enhanced biosecurity measures, such as BSL-3, especially if the specimen is thought to be HPAI. The assay also requires highly trained technical staff to perform and has the longest turnaround time of any of the AI viral detection tests and diagnostics, which can extend up to 2 weeks or more depending on whether multiple passages are used, how quickly the virus grows to a high titer, and if subsequent testing for characterization is required.

### 2.4. Challenges in Existing Diagnostic Practices

Despite the significant advancements in molecular and serological assays for AI, several challenges remain to effectively diagnose and control outbreaks in a timely manner. One major disadvantage is the reliance on centralized laboratory infrastructure for all of the tests provided. The advanced biosafety facilities, highly skilled personnel, and sophisticated equipment required for gold standard diagnostics, such as virus isolation and RT-qPCR, are typically only found in regional or national reference laboratories [31,52]. Usually, this centralized method causes delays in diagnosis, especially in rural or low-resource environments where outbreaks are frequent. These time lags may significantly reduce the window for effective containment, potentially resulting in further viral spread in commercial and backyard poultry populations.

When it comes to AI outbreaks, they are most often in the field in areas with restricted access, making the issue of the lack of point-of-care (POC) diagnostic options a glaring concern. Even with the growing development of lateral flow assays and rapid antigen tests in research, many still have poor subtype specificity and low sensitivity, especially when used for early or asymptomatic infections [53]. Furthermore, environmental factors like temperature, humidity, and sample quality and handling often impair assay performance in field settings. Without ruggedized, user-friendly platforms for on-site testing, poultry producers continue to rely on sample shipment and centralized reporting, endangering the efficacy and biosecurity of outbreak response.

Additionally, many conventional diagnostic approaches are incompatible with DIVA strategies. DIVA-compliant surveillance is critical in regions where vaccination is implemented to control LPAI or mitigate HPAI risks [54]. However, according to the vaccine platforms currently under study, serological tests, such as HI and ELISA, frequently identify antibodies against internal or conserved viral proteins that are present in both infected and vaccinated birds. This hinders disease surveillance and trade compliance by making it challenging to differentiate between exposure and immunization. In many endemic areas, DIVA-compatible recombinant vaccines and companion diagnostics are still not commonly used due to their high cost, regulatory barriers, and limited availability. The best approach would be a more thorough one in which vaccines are developed using DIVA techniques in diagnostic testing [17].

Altogether, these challenges underscore the urgent need for decentralized, sensitive, and DIVA-compatible diagnostic technologies when it comes to AI disease control and prevention in the face of growing epidemics and pandemics globally. Such innovations would enable real-time surveillance, facilitate targeted interventions, and support sustainable control programs across diverse poultry production systems in a more timely and economically feasible fashion.

## 3. Biosensor Technologies: Fundamentals and Classifications

The rapid emergence and spread of avian influenza viruses, such as H5N1 and H7N9, underscore the urgent need for swift, accurate, and reliable detection methods. Traditional laboratory-based diagnostics, such as reverse transcription–polymerase chain reaction (RT-PCR) and viral culture, while sensitive, often suffer from limitations in turnaround time, the requirement for sophisticated equipment, and the need for skilled personnel. These limitations can hinder timely decision making during outbreaks.

In contrast, biosensors offer promising alternatives owing to their potential for real-time, on-site diagnostics, high sensitivity, and specificity. The ability to detect viral components directly from complex biological samples will make them invaluable tools in managing outbreaks allowing for rapid screening, early warning, and timely interventions. For field use, biosensors are expected to operate through matrix effects (mucus, proteins, surfactants) and environmental stressors (temperature swings, dust, vibration) while maintaining clinically relevant LOD and acceptable time to result.

### 3.1. Definition and Components of Biosensors

According to the International Union of Pure and Applied Chemistry (IUPAC), a biosensor is an analytical device that combines a biological recognition element with a transducer to produce a measurable signal directly proportional to the concentration of a target analyte. All biosensors are fundamentally composed of three integrated components working synergistically: (1) a biorecognition element that selectively binds the analyte, (2) a transducer that converts this binding event into a physical signal (electrical, optical, acoustic, thermal), and (3) a signal processing/readout unit that transforms the signal into a quantitative output, such as concentration or presence/absence. Understanding these fundamental components is essential for grasping the operational principles and potential applications of biosensors.

#### 3.1.1. Biorecognition Elements

The biorecognition element is responsible for specific interaction with the target analyte, providing selectivity to the biosensor. For AIV detection, biosensors typically target either viral nucleic acids (RNA), viral proteins, such as HA and NA, or whole virions. The choice of target directly governs selectivity (broad influenza screening vs. subtype-specific identification), matrix tolerance, and overall feasibility for deployment under real field conditions.

Biorecognition elements used in AIV biosensors broadly include (i) antibodies, including monoclonal antibodies for subtype-specific HA recognition and polyclonal antibodies for broader antigen capture; (ii) nucleic acid probes that hybridize to influenza RNA targets; (iii) aptamers, which are synthetic single-stranded oligonucleotides engineered for high-affinity binding to viral proteins or whole virions; (iv) host-mimetic ligands (e.g., sialic-acid-based binding layers) that exploit conserved influenza attachment mechanisms; and (v) synthetic receptors, particularly molecularly imprinted polymers (MIPs), which create template-shaped binding sites resembling “plastic antibodies.” Importantly, these recognition strategies can support different biosensor archetypes, including immunosensors, aptasensors, nucleic acid biosensors, and MIP-based sensors, often coupled with electrochemical, optical, or piezoelectric transducers. Examples include antibodies [55] targeting influenza hemagglutinin or neuraminidase proteins, nucleic acid probes complementary to viral RNA [56,57] or DNA [58], or aptamers engineered for high affinity and specificity [59]. For instance, immobilized monoclonal antibodies against H5N1 hemagglutinin have been used in immunosensors for bird flu detection. Meanwhile, nucleic acid probes and isothermal amplification primers enable sensitive detection of viral RNA and are commonly used in microfluidic biosensor platforms for subtyping (e.g., H5/H7/H10) [60].

The selection of biorecognition elements is critical for maintaining high specificity in complex AIV-relevant matrices, such as cloacal/oropharyngeal swabs, feathers, litter, bird droppings, and environmental water or wastewater. These sample types contain abundant interferents—including background proteins, mucus components, particulate matter, and inhibitory compounds—that can promote nonspecific adsorption and surface fouling, suppress recognition interactions, and ultimately cause signal drift or misclassification. Accordingly, biorecognition element selection must prioritize not only binding affinity and subtype selectivity but also matrix tolerance, chemical stability, and robust performance across temperature and humidity fluctuations typical of field deployment. Importantly, the optimal recognition strategy depends strongly on the intended use case: nucleic-acid-based recognition (probes and amplification primers) can provide the highest analytical sensitivity and subtype discrimination, whereas protein- or whole-virion recognition enables faster workflows that are well-suited to rapid screening and on-site surveillance. In practice, the biorecognition layer often becomes the limiting factor that separates proof-of-concept laboratory performance from reliable real-world diagnostics. Therefore, successful AIV biosensor design requires coordinated optimization of the biorecognition element, immobilization chemistry, and a transduction method to ensure both analytical performance and operational usability in field settings. A detailed discussion of biorecognition elements can be found in Section 4.

#### 3.1.2. Transducers

The transducer converts biological interaction into a measurable signal. It transduces the biochemical event into an electrical, optical, mechanical, thermal, or magnetic signal. Transducers can be classified based on their mode of signal conversion, such as electrochemical, optical, piezoelectric, thermal, or magnetic transducers, each suited for specific applications and analytes. For example, in electroanalytical biosensors, the binding of a virus to an antibody on an electrode surface may result in a change in electrical impedance, which can be measured directly. Optical sensors, on the other hand, might detect shifts in light properties, such as refractive index changes in surface plasmon resonance (SPR) systems, enabling label-free, real-time monitoring of viral binding.

#### 3.1.3. Signal Processing Systems

Once the transducer generates a signal, signal processing systems amplify, filter, and convert this signal into a quantifiable and interpretable form. This involves electronic circuitry, data acquisition systems, and, sometimes, digital processing algorithms. The processed signal correlates with the analyte concentration, enabling quantitative analysis.

A popular current trend is the integration of signal conditioning and readout functionalities into handheld devices and smartphones. This integration enables automatic baseline stabilization, calibration, and advanced data analytics, ultimately delivering user-friendly, decision-ready results in real time.

### 3.2. Classification Based on Signal Transduction

Biosensors can be broadly categorized based on the type of signal transduction mechanism they employ. This classification reflects the diverse physical principles harnessed for analyte detection, each with its own advantages, limitations, and suitable applications.

#### 3.2.1. Electroanalytical Biosensors

Electroanalytical biosensors quantify changes in electrical properties, such as current, potential, charge transfer resistance, or capacitance. These platforms are frequently used for POC testing due to portability, low power requirements, compatibility with miniaturized electronics, and relatively low cost. There are the following types of electroanalytical sensors.

Amperometric: Measures the change in current (rate of electron transfer) resulting from the oxidation or reduction of the electroactive species. The classic glucose biosensor operates on this principle.

Potentiometric: Measures the change in potential (voltage) at zero current. This is often used with ion-selective electrodes (ISEs) to detect ions or charged species.

Conductometric/impedimetric [61]: Measures the change in electrical conductance or impedance of the medium between electrodes, typically due to the consumption or production of ionic species or an alteration in the charge transfer pathway.

For AIV, electroanalytical sensors have been reported using antibodies, aptamers, or host-mimetic ligands to capture virus particles or HA proteins, followed by signal readout through EIS or other methods. For example, an electrochemical immunosensor employing electrodes coated with anti-influenza antibodies can detect viral particles via changes in impedance [62]. When anti-HA antibodies or HA-binding aptamers are immobilized on gold or carbon electrodes, virion binding perturbs interfacial electron transfer and double-layer properties in ways that EIS or differential pulse voltammetry can quantify without labels; enzymatic cycles (e.g., horseradish peroxidase) or redox mediators can be added when further amplification is required. These sensors are particularly useful for POC testing in field conditions, such as in poultry farms, because they can be integrated with handheld readers and smartphone-based interfaces. Electroanalytical sensors have become the workhorses of portable pathogen sensing because they combine high sensitivity with low power draw and straightforward miniaturization.

#### 3.2.2. Optical Biosensors

Optical biosensors utilize the properties of light to detect biological interactions, such as absorbance, fluorescence, luminescence, or refractive index, following the biological recognition event. They are widely employed due to their high sensitivity and their ability to achieve real-time kinetic monitoring, although instrumentation costs can be higher than electroanalytical formats depending on system complexity. Two major categories are label-free refractometric platforms and label-based platforms.

Surface plasmon resonance sensors [63,64] and related refractometric methods comprise a label-free technique that detects changes in the refractive index at a metal–dielectric interface due to biomolecular binding, offering high sensitivity for kinetic studies. By providing real-time kinetic readouts of HA–receptor interaction, SPR supports affinity characterization and subtype differentiation without secondary reagents. For example, SPR can observe real-time binding of viral proteins without the need for labels, providing a high-sensitivity, label-free detection platform.

Fluorescence and chemiluminescence-based sensors, meanwhile, involve labeling the analyte or receptor with a fluorophore and measuring the resulting light emission upon binding or reaction, thus enabling highly specific detection even amidst complex sample matrices. Similarly, colorimetric readouts—often nanoparticle-amplified—enable visually interpretable lateral-flow-like formats or phone camera quantitation for AIV RNA and antigen detection.

Representative AIV optical biosensors have leveraged SPR for both Direct Antigen/virion sensing and for measuring HA–receptor affinity. For example, aptamer- or antibody-functionalized SPR chips have been reported for H5N1 detection with rapid label-free readout and potential for near-real-time monitoring [65,66]. In addition, nanoparticle-enhanced fluorescence/FRET formats using host-mimetic ligands (e.g., sialic acid analogs) enable broad influenza capture with portable optical readout modules, supporting farm-level screening when subtype resolution is not required [67].

#### 3.2.3. Piezoelectric and Acoustic Sensors

Piezoelectric and acoustic sensors—quartz crystal microbalance (QCM) [68] and surface acoustic wave (SAW)—convert mass loading and viscoelastic changes at a functionalized surface, such as due to viral particles binding to antibodies, into frequency or phase shifts. QCM measures the change in the resonant frequency of a piezoelectric quartz crystal. When mass is added to or removed from the crystal surface (due to analyte binding), the frequency shifts proportionally. Microcantilevers use highly sensitive structures that can detect mass changes by measuring the change in their physical deflection or resonant frequency. These sensors are highly sensitive to larger biomolecules and can operate in real time. However, viscosity and temperature variations introduce damping and baseline wander; practical designs therefore integrate thermal control, microfluidic flow conditioning, and mechanical isolation.

QCM-based AIV sensors commonly employ antibodies, aptamers, or synthetic biorecognition elements, such as molecularly imprinted polymers (MIPs), to capture whole virions or HA, producing measurable frequency shifts proportional to mass loading. Hydrogel or nanoparticle amplification layers have been used to improve capture efficiency and extend sensitivity in complex matrices [68,69]. Notably, influenza MIP layers integrated with QCM have also been explored for subtype-discriminating binding profiles, illustrating the potential of robust “plastic antibody” recognition for field surveillance [MIP-QCM] [70].

#### 3.2.4. Thermal and Magnetic Sensors

While less common, thermal and magnetic biosensors also contribute to the detection landscape. Thermal (calorimetric) sensors quantify minute heat fluxes from enzymatic amplification or immunoreactions, allowing for indirect detection (i.e., exothermic steps in enzyme-linked amplification) when optical/electroanalytical routes are impractical. Magnetic biosensors use magnetic nanoparticles (MNPs) conjugated to probes/antibodies; changes in magnetization, relaxation, or magnetoresistance are robust in turbid samples and pair naturally with magnetic pre-concentration to boost LOD.

### 3.3. Analytical Performance Metrics

The performance of any biosensor hinges on several key metrics, including sensitivity (slope) and LOD (e.g., virions/mL or copies/µL), specificity (against co-circulating AIV subtypes and confounders), dynamic range, time to result, matrix tolerance (e.g., feathers vs. swabs), reproducibility/robustness, and portability/throughput (single-use vs. multiplex).

Sensitivity determines how low a concentration of viruses can be detected—a crucial factor for early diagnosis. Specificity ensures that the sensor responds solely to the target virus, avoiding false alarms caused by other microorganisms or substances. The LOD defines the smallest quantity reliably identified, while response time indicates how quickly results can be obtained, a vital aspect during outbreaks. Portability and ease of use also determine whether a biosensor can be effectively deployed in field conditions, making it a practical tool for rapid disease surveillance. For AIV surveillance, practical benchmarks target (i) ≤30–45 min total assay time, (ii) sub-clinical detection (low viral load), (iii) minimal sample prep, and (iv) compatibility with on-farm workflows and DIVA strategies.

### 3.4. Comparison of Biosensor Platforms

Across platforms, electroanalytical formats typically offer the best blend of sensitivity, simplicity, and low cost for decentralized screening; optical platforms excel in kinetics and label-free operation for confirmatory or higher-complexity testing; acoustic sensors provide label-free, whole-particle mass sensitivity that benefits virion-level capture; and thermal/magnetic approaches add rugged options for challenging matrices. Selection is ultimately governed by the required LOD/time-to-result, the intended sample matrix (swab, feather, litter, milk/wastewater), and constraints on cost, power, and user training. A comparison of AIV biosensors is illustrated in Table 2.

## 4. Biorecognition Elements in Avian Influenza Biosensors

Biosensors have emerged as an alternative rapid POC diagnostic tool to conventional methods to precisely identify the causative pathogen. Biosensors are highly advantageous due to their high sensitivity (few false negatives), specificity (few false positives) [76], low cost, ease of use, and rapid response time for analyte detection [77].

A biosensor comprises a biorecognition element (bioreceptors) that specifically binds to a target (bio)analyte (specific surface molecules (e.g., biomarkers), such as proteins and epitopes or nucleic acids) and transducer elements that translate the binding occurrence into a detectable signal with a detector providing the readout [78,79]. An ideal biorecognition element possesses a selective and potent affinity towards the bioanalyte, thus endowing a biosensor with good specificity [79].

**Figure 3:**
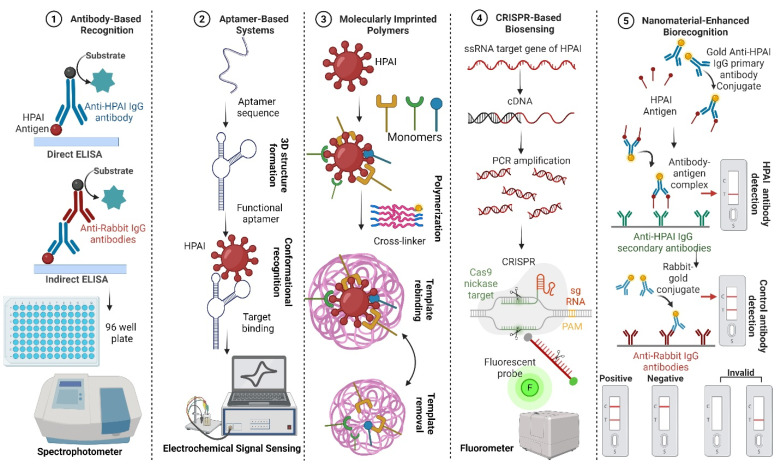
Biorecognition elements in avian influenza biosensors. This figure illustrates five major biorecognition strategies used in avian influenza virus (AIV) detection platforms. 1—Antibody-Based Recognition: Utilizes monoclonal or polyclonal antibodies in formats such as direct and indirect ELISA. Target antigens from highly pathogenic avian influenza (HPAI) interact with enzyme-linked antibodies, producing a measurable signal upon substrate addition, typically read by a spectrophotometer in a 96-well plate. 2—Aptamer-Based Systems: Single-stranded DNA aptamers fold into specific 3D conformations capable of binding HPAI targets with high specificity. This binding induces conformational changes, generating electrochemical signals that are recorded via electrochemical sensors. 3—Molecularly Imprinted Polymers (MIPs): Synthetic receptors are fabricated by polymerizing functional monomers around the HPAI virus. After removal of the template, the MIP retains selective binding sites, allowing for target rebinding based on shape and functional complementarity. 4—CRISPR-Based Biosensing: Involves the conversion of HPAI viral RNA into complementary DNA (cDNA), followed by amplification. CRISPR-Cas systems (e.g., Cas9 nickase) recognize specific sequences using guide RNA (sgRNA) and produce a signal, often via fluorescent probes, detected by fluorometers. 5—Nanomaterial-Enhanced Biorecognition: Leverages gold nanoparticle conjugates for colorimetric detection. Gold particles conjugated to IgG antibodies bind with HPAI antigens in patient samples. Results are visualized using lateral flow assays (LFAs), with control lines ensuring test validity. Interpretations include positive, negative, and invalid outcomes.

Bioreceptors are mainly classified as natural and artificial [80,81,82]. Natural biorecognition elements include antibodies, nucleic acids, and enzymes, originate from living organisms, and are harvested in laboratories. Artificial bioreceptors, on the other hand, are either fully/partially synthesized or engineered from natural biorecognition elements [82,83]. They primarily consist of aptamers, MIPs, and recombinant natural bioreceptors (like antibody fragments). Recent advances in AIV biosensing leverage diverse biorecognition elements, as illustrated in Figure 3.

The measurement strategies used for the diagnosis of viral infections are categorized as (i) direct pathogen detection (viral surface proteins or the virus itself) [84,85,86], (ii) recognizing pathogen-associated viral genetic material, such as nucleic acids (either viral RNA or DNA) [85,86,87,88,89,90,91], or (iii) indirectly detecting the biomarkers that are present as a result of the specific host immune response, including antibodies [85,86,87,88,89,90,91]. Table 3 summarizes the difference between the Direct Antigen and Indirect Antibody detection tests.

### 4.1. Antibody-Based Recognition

The antibodies’ immunoglobulins (Ig) are large glycoproteins produced by white blood cells with strong affinity and specificity towards their target analytes. These qualities make them a natural and popular choice as biorecognition elements and, consequently, they have been adapted for use in pathogen identification as capture probes in sensor platforms [79].

Although antibodies have been the precise and widely used gold standard biorecognition elements for decades due to their exceptionally high affinity and specificity towards their target analytes due to their inherent abilities for antigen detection, their limitations include poor stability, reproducibility (difficulties scaling up), and high cost in large-scale production [92], which complicate their use in sensing platforms that require a long shelf-life. Other problems relate to lengthy production time and the need for ethical approval, which increase costs [93]. Therefore, research in recent years has also focused on finding alternate biorecognition elements with improved specifications. The most significant direct and indirect serological assays are based on chemiluminescent assays [94], ELISA [95], and lateral flow immunoassays (LFA) [96].

#### 4.1.1. Lateral Flow Immunoassays (LFA)

Direct pathogen measurement is performed exclusively through antibody–antigen interaction [85,86,87,88,89,90,91] by targeting the virus-specific proteins [84,85,86] (nucleocapsid, surface, or transmembrane proteins) of a target pathogen. The sensitivity and specificity of the detection of antigens are 50–80% and 90%, respectively [97].

LFAs in the form of rapid antigen tests using virus-specific antibodies are routinely used for the (semi-)quantitative or qualitative detection of various analytes in clinical settings due to their low cost, instrument-free use, easy applicability, short turnaround times (5–30 min), and highly favorable use for on-site virus detection to control infectious disease spread to a certain extent [98].

In brief, LFAs consist of a conjugation pad, which has antibodies temporarily fixed to the surface [96]. A mixed sample is then placed on the sample pad, which directs sample flow to the conjugation pad, where the conjugated antibodies bind the analyte (or antigen) of interest. The analyte–antibody mix subsequently migrates along a membrane through capillary flow across test and control strips. These strips are coated with antibodies detecting the analyte of interest, and a positive test is confirmed by a color change in control and test lines [96,99,100].

#### 4.1.2. Enzyme-Linked Immunosorbent Assays (ELISA)

ELISA has the advantages of high sensitivity and specificity compared with the detection of the microneutralization method (HA and HI) [101,102], but the ELISA detection method has poor reproducibility [103].

ELISA to directly detect H5 antigen provide strong evidence of H5 avian influenza virus infection [104]; however, they are not widely used for human influenza diagnostics, which also requires concentrated and purified live virus preparations to be used as test antigens [105,106,107].

Monoclonal antibody (Mab)-based diagnostic antigen detection tests use a homogenous population of antibodies derived from a single antibody-producing cell whereby all antibodies produced are identical and of the same specificity for a given epitope [108], providing a basis for an effective diagnostic reagent [109]. However, using one single Mab for H5 AIV antigen detection, in most cases, will not cover all of the H5 subtypes of AIV circulating around the world due to mutated antigenic epitopes during the evolution of AIV as an RNA virus.

ELISA can detect cross-reactions between different subtypes of AIV [110,111]. Double-antibody sandwich ELISA (DAS-ELISA) uses monoclonal antibodies (MAb) as capture antibodies and horseradish-peroxidase-labeled rabbit-derived polyclonal immunoglobulin G (IgG) as detection antibodies; they can detect H1–H15 subtypes of AIV nucleoprotein (NP) and exhibit no cross-reaction with other avian pathogens [112].

Antigen capture ELISA (AC-ELISA) is based on H5 HA-specific monoclonal-antibody-targeting conformational epitopes and N1 NA linear epitopes, which can concurrently detect H5 and N1 subtype antigens; it is used for the rapid detection of H5N1 AIV [113].

More than a couple of monoclonal antibodies or polyclonal antibodies are required to reach appropriate specificity and sensitivity of detection, which increases production cost [104]. The antigen-capture dot ELISA (H5-Dot ELISA) detection method is an attempt to make a universal H5 AIV rapid detection test for the detection of the H5 subtype AIV using two complementary Mabs, Mab 6B8 and Mab 4C2, to identify arginine and lysine at position 189 of H5N1 AIV, as well as asparagine and serine at position 155. The HA protein of H5N1 AIV contains a specific polybasic cleavage site rich in arginine [114]. The specificity of the optimized dot ELISA was examined by using 100 H5 strains, including H5N1 HPAI strains from multiple clades (including clades 0, 1, 2.1, 2.2, 2.3, 4, 7, and 8), 36 non-H5N1 viruses, and 4 influenza B viruses. No cross-reactivity was observed for any of the non-H5N1 viruses tested. The LOD of the test varied from 0.5 to 0.125 HA units/200 µL of the sample. The sensitivity of the dot ELISA kit was able to detect the presence of the virus at a concentration down to 105 EID50/mL in swabs, which is more sensitive than the titer of virus (108 EID50/mL) shed from non-symptomatic birds [115].

To date, many indirect ELISA detection methods have been developed to indirectly detect antibodies to the AIV in poultry, which is essential for epidemiological investigations [116], as well as other species, including humans [117]. Most of them require specific anti-immunoglobulin conjugates.

Competitive ELISA (CELISA) is based on the recombinant baculovirus of AIV for the serological diagnosis of AIV in various birds. This CELISA does not require the use of several anti-immunoglobulin conjugates or live viruses, avoiding the potential risk of infection from exposure to live viruses [118]. Epitope-blocking ELISA (EB-ELISA) can be used to detect specific antibodies to H5N1 AIV in human or animal serum. This detection method relies on a Mab that can bind to the epitope of H5 hemagglutinin. The EB-ELISA can easily detect H5N1 antibodies in the sera of immunized animals or convalescent humans and has 100% specificity [102].

The U.S. Food and Drug Administration (FDA) regulates a variety of in vitro diagnostic (IVD) tests for detecting influenza A and B viruses, including rapid antigen tests and serological tests. These tests play a critical role in clinical decision making, outbreak management, and public health surveillance [119]. Table 4 shows key comparison criteria of the selected FDA-cleared influenza antigen tests.

### 4.2. Aptamer-Based Systems

Recent research has been more focused on artificial biorecognition elements, such as aptamers, also known as “chemical or artificial antibodies” [120], which can be modified to improve their biorecognition capabilities, and creating low-cost biosensors on a large scale while being arguably more stable than antibodies [79,83].

Aptamers are short ssDNA or ssRNA molecules, having a length of 25–100 bases, that fold into stable three-dimensional conformations. They are selected through a high-flux screening method named “Systematic Evolution of Ligands by Exponential Enrichment (SELEX)” [65]. The resultant aptamer achieves optimum interaction with the target analyte of interest and is easily distinguished after repetitively processing binding, washing, and amplification steps through SELEX [120,121].

The aptamer sequence binds to targets via hydrogen bonding, van der Waals forces, and/or electrostatic interactions [122,123,124] with high affinity and specificity to an extensive variety of the target of interest, from small molecules to big proteins and also live cells, such as whole bacteria [125].

Aptamers exhibit better performance than monoclonal antibodies, particularly for virus detection, to build aptasensors. The reason can be summarized as follows: (i) reproducible and cheaper chemical production methods, which do not require living organisms as raw materials; (ii) an easy selection procedure, which does not depend on a particular analyte; (iii) high specificity between different virus genotypes due to the elimination of cross-reaction during SELEX; (iv) high affinity and selectivity toward the target analyte; (v) high thermal and chemical stability; (vi) ease in modification and functionalization and labeling without loss of function; and (vii) lower molecular weight, nontoxicity, and low immunogenicity [65,120,122,126,127].

Despite aptamers’ numerous advantages, the general unfamiliarity regarding aptamers and their interesting performance [128] slow the translation of aptamer-based products to relevant POC diagnostics [129,130]. In addition, they require resource-intensive and time-consuming initial development, including multiple steps for their design, characterization, and optimization [79,83]. Table 5 summarizes aptamer-based methods for detecting influenza viruses.

### 4.3. Molecularly Imprinted Polymers (MIPs)

Artificial material-based biorecognition elements, known as MIPs, rely on the specific morphology or shape of the target for selective capture due to the tailor-made binding sites, which fully complement the template molecules in size, shape, and functional groups [134,135].

The most common examples of MIPs are cell imprinted polymers (CIPs), which are widely applied in the detection of the virus in diagnostics [136].

MIPs are label-free bioreceptors with a high specificity due to the interaction with the whole viral particle compared with ligand-based biorecognition platforms as native antibodies that interact only with its receptor site. However, during a binding event between the template and the molecules, conversion of the resulting signal by detection systems needs to be improved, in addition to elimination of noise in the signal.

Recent studies illustrate the application of virus-imprinted polymers (VIPs) and molecularly imprinted biosensors in viral diagnostics. One study introduced a VIP capable of selectively recognizing and immobilizing H1N1 influenza A virus in a multichannel microfluidic sensor, leveraging its electrochemical properties to achieve high sensitivity and specificity in swine samples [137]. Similarly, a novel potentiometric biosensor employing molecularly imprinted self-assembled monolayer films enabled rapid differentiation between virus strains with minimal sample volumes. This approach allowed for precise detection of influenza A virions and SARS-CoV-2 spike proteins while significantly reducing detection time, demonstrating the potential of MIP-based platforms for fast, accurate, and label-free viral detection [138].

### 4.4. CRISPR-Based Biosensing

Clustered regularly interspaced short palindromic repeats (CRISPR) are a family of DNA sequences originating in bacteriophages that have previously infected prokaryotes and subsequently become incorporated into their genomes. Several applications, including gene editing [139,140] and genome imaging [141], have been used in conventional approaches to quickly, simply, and efficiently transform endogenous genes into a wide variety of cell types and in organisms to manipulate them genetically [142]. Lately, many efforts have been directed towards understanding their potential for diagnostic applications [143]. CRISPR-Cas systems have been programmed to sense any specific DNA or RNA of interest, such as pathogenic genetic material in nucleic-acid-based diagnostics [144,145,146,147].

Cas is a protein associated with endonuclease activity that recognizes and cleaves target nucleic acid sequences like a pair of scissors [148,149]. It is a viable tool when adapted for pathogen detection. Among DNA-targeting Cas proteins, Cas9 enzyme guided by single-guide RNA (sgRNA) can specifically bind to target double-stranded DNA, cleave it, and eventually result in its breakage [150,151].

An alternative class of Cas protein, Cas12, is a proficient enzyme that possesses the *cis*–*trans* cleavage activity of ssDNA to create staggered cuts in dsDNA. It is being used for pathogen detection [66]. For example, a DNA endonuclease targeted CRISPR trans reporter (DETECTR) method was developed based on the activation of Cas12a ssDNase.

An extremely sensitive and portable platform method for DNA or RNA detection from real clinical samples was named specific high-sensitivity enzymatic reporter unlocking (SHERLOCK). This platform is able to detect multiple targets through the use of a multiplex fluorescence-based detection system [152]. Table 6 summarizes CRISPR-based methods for detecting influenza viruses.

### 4.5. Nanomaterial-Enhanced Biorecognition

The lateral flow immunochromatography technique uses capillary action to move the tested antigen from the sample pad at one end of the test strip to react with gold nanoparticle-labeled antibodies on the binding pad. The antigen–antibody complex formed by the binding of the gold-labeled antibodies gathers on the detection line, and the color development results can be observed by the naked eye [157].

Rapid semi-quantitative detection of H5 AIV was designed to reflect the degree of virus infection in different organs [158]. It uses a colloidal gold immunochromatographic technique and four test lines with different concentrations. Cysteamine–gold-coated carboxylated fluorescent europium nanoparticles have also been developed for rapid detection of the H5N1 virus [159]. The potential surface charge and fluorescence stability of such fluorescent nanomaterials can improve the detection limit by about eightfold. Moreover, sandwich hybridization of quantum dots and magnetic beads has been established as a new method for the detection of H5N1 AIV [160] in which quantum dots were coupled with probes to generate the fluorescent signals while magnetic beads were used to separate and concentrate signals.

In general, the lateral flow chromatography strip constitutes an alternative and cheap diagnostic tool in the clinical field, especially in developing countries [161]. However, the examiner can only complete simple technical operations and lacks the scientific information necessary to rationally evaluate the clinical value and reliability of the test results and the detection method [162]. Table 7 summarizes nanomaterial-based methods for detecting influenza viruses.

## 5. Targeting Viral Subtypes and Pathogenicity Markers

### 5.1. Hemagglutinin (HA) and Neuraminidase (NA) Subtype Detection

Influenza A viruses (IAVs) are classified into subtypes according to the antigenic characteristics of their two major surface proteins, HA and NA. So far, researchers have identified 16 HA (H1–H16) and 9 NA (N1–N9) subtypes, most of which circulate in wild waterfowl—the natural reservoirs of these viruses [173,174]. Although reassortment between HA and NA segments could theoretically produce a wide range of subtype combinations, only a fraction of these have actually been detected in nature [175]. Identifying the correct subtype is essential for understanding which strains are circulating, tracking reassortment events, and monitoring the emergence of new or unusual influenza viruses.

For many years, serological assays, such as HI and NI, have been considered the gold standard for determining HA and NA subtypes. These tests rely on the specificity of antigen–antibody interactions and continue to play an important role in validating modern diagnostic tools [176]. However, serological approaches come with several drawbacks. They depend on high-quality reference antisera, can be labor-intensive, and often take considerable time to complete. Their sensitivity may also be limited when virus levels are low or when antigenic drift reduces the ability of antibodies to recognize circulating strains [176,177].

Because of these challenges, molecular diagnostic methods have become increasingly central to influenza surveillance. Techniques such as RT-PCR, RT-qPCR, LAMP, and NGS offer rapid, sensitive, and highly specific detection of HA and NA subtypes, including high-consequence H5 and H7 viruses [178,179,180,181]. Beyond subtype identification, molecular assays can reveal genetic markers linked to pathogenicity, host adaptation, and virulence, providing critical information for early warning systems. These approaches complement traditional serology while offering the speed and precision needed for modern outbreak response, enhanced surveillance, and public health preparedness.

Accurately identifying HA and NA subtypes—especially the high-risk H5 and H7 types—is crucial for effective avian influenza surveillance and control. Subtyping plays a direct role in early outbreak detection, risk assessment, and the implementation of targeted responses. It also helps researchers understand the broader ecology and evolution of influenza viruses, which show remarkable diversity in wild birds and other natural reservoirs [174,182].

Among all subtypes, H5 and H7 viruses remain the most concerning because they can evolve into HPAI strains capable of causing severe disease in poultry and, in some cases, infecting humans [17,183]. Regular monitoring of these subtypes allows authorities to quickly detect emerging variants and take prompt action—whether that involves quarantine, depopulating infected flocks, or adjusting trade policies. Subtype data are also essential for guiding vaccination programs, ensuring that vaccines are well-matched to circulating strains [17,184].

More broadly, routine subtyping strengthens molecular epidemiology efforts by helping track viral evolution, identify reassortment events, and flag newly emerging subtypes that may carry pandemic potential [174,182]. When subtype information is incorporated into national and international surveillance networks—such as those led by World Health Organization (WHO), OIE/WOAH, and FAO—it supports coordinated reporting, timely risk communication, and more effective global preparedness [52,185].

### 5.2. Distinguishing LPAI from HPAI

In wild waterfowl, AIV infections are usually asymptomatic and do not cause noticeable tissue damage [186,187]. The virus primarily replicates in the gastrointestinal tract (GIT), leading to significant fecal shedding and only minimal oropharyngeal shedding [188,189,190]. As a result, fecal–oral transmission is the main route through which AIVs spread among wild birds.

When AIVs move from wild waterfowl into terrestrial poultry, such as chickens and turkeys, transmission often occurs through direct or indirect contact [191,192]. In these hosts, viral replication is mostly restricted to the GIT and the respiratory tract (RT), usually causing only mild disease. Viruses that cause this limited pathology are classified as Low Pathogenic Avian Influenza Viruses (LPAIVs) [193]. However, LPAIVs of the H5 and H7 subtypes can evolve into Highly Pathogenic Avian Influenza Viruses (HPAIVs) when introduced into poultry populations [193].

This transition from LPAIV to HPAIV is driven by the acquisition of basic amino acids at the HA cleavage site, forming a multibasic cleavage site (MBCS)—the key molecular feature associated with high virulence in poultry. These changes can arise through point mutations, sequence duplications, or non-homologous recombination (NHR) between viral or host RNA and HA RNA. The MBCS allows the HA protein to be cleaved by proteases found throughout the host’s tissues, enabling systemic infection, whereas LPAIV HA cleavage is restricted to certain tissues.

The OIE provides the official criteria for distinguishing LPAIVs from HPAIVs [194]. Classification relies on two main factors. The first is the Intravenous Pathogenicity Index (IVPI), determined by inoculating ten 4–8-week-old chickens intravenously and monitoring them for 10 days. Birds are scored every 24 h: 0 if normal, 1 if sick, 2 if severely sick, and 3 if dead. The IVPI is calculated as the average score per bird per observation, and viruses with an IVPI of 1.2 or higher are classified as HPAIV. The second factor is the HA cleavage site sequence. Viruses with an MBCS identical to that of previously known HPAIVs are considered highly pathogenic regardless of IVPI. For viruses with a novel MBCS motif, IVPI testing is required to confirm their pathogenicity.

### 5.3. Multiplexed Detection Approaches

Recent advances in diagnostic technology have led to the development of multiplexed, microfluidic “lab-on-a-chip” platforms that can detect and differentiate multiple AIV subtypes at the same time. For example, a 2025 study demonstrated a microfluidic device capable of distinguishing H5, H7, and H10 subtypes in a single assay using subtype-specific primers and probes targeting conserved regions of the HA gene, combined with a recombinant enzyme polymerase amplification (RPA) system [60]. This platform achieved remarkable sensitivity, detecting as few as two viral copies per reaction, and produced results fully consistent with standard quantitative PCR when tested on 100 clinical samples [60]. By consolidating multiple tests into one assay, this approach significantly reduces time, labor, and the risk of contamination compared with running separate PCRs for each subtype.

While identifying subtypes is essential, the ability to integrate pathogenicity profiling into multiplexed assays offers a major advance for surveillance and outbreak response. The modular design of the microfluidic RPA platform could, in principle, be adapted to include probes or primers that detect molecular markers of high pathogenicity, such as MBCS in the HA gene or other known virulence factors. Other multiplex platforms, including isothermal or real-time RT-PCR assays, have also been developed for concurrent subtype detection. For instance, a multiplex TaqMan real-time RT-PCR assay has been used to detect H4, H6, and H10 subtypes simultaneously, demonstrating the feasibility of multi-subtype diagnostics in surveillance programs [195].

Overall, these multiplexed platforms—which combine subtype identification with potential pathogenicity marker detection—represent powerful tools for rapid, field-deployable influenza surveillance. They not only provide information on which subtypes are circulating but, with further development, could also highlight strains of greatest pathogenic concern. Such capabilities are particularly valuable for outbreak preparedness and response, especially in low-resource or field settings where timely and accurate detection is critical.

## 6. Differentiating Infected from Vaccinated Animals (DIVA)

DIVA is pivotal for effective and sustainable avian influenza control, shaping outbreak responses, surveillance accuracy, and international trade policy. Vaccination strategies are being studied and created for potential widespread use, especially in areas labeled as high-risk or dealing with ongoing HPAI circulation. This makes it increasingly important to tell the difference between natural infection and vaccine-induced immunity. Traditional DIVA-compatible tests, despite being useful, still have significant issues with sensitivity, antigenic drift, and use in the field. New biosensor-based methods are emerging to make use of new biorecognition elements, multiplexing features, and quick on-site analysis to improve DIVA performance. This section will highlight the importance of DIVA frameworks alongside vaccine design, examine shortcomings of existing tools, and explore promising strategies and experimental systems.

### 6.1. Importance of DIVA in Outbreak Management and Trade

DIVA is a cornerstone of modern avian influenza control, as this strategy aims to preserve the epidemiological value of surveillance of the pathogen while allowing for strategic vaccine use to limit disease spread. In an outbreak setting, DIVA-compatible approaches enable authorities to detect field infection in vaccinated flocks, identify residual transmission chains, apply targeted control measures, and document immune escape, all without assuming that all seropositive birds reflect natural infection. The capacity to distinguish vaccine-induced seropositive results from infection-derived responses reduces the risk that vaccinated but infected flocks remain undetected. This concern is paramount, as imperfect vaccine-induced protection can allow for silent or subclinical circulation of avian influenza [196,197].

Beyond on-farm outbreak control, DIVA has direct implications for maintaining international trade and reliable market access. Countries that implement broad vaccination programs without validated DIVA strategies have historically faced trade restrictions or loss of disease-free status on an international scale due to importers and international frameworks requiring confidence that all exported birds and their products are free from avian influenza infection [198]. A well-validated DIVA system would, therefore, provide a strategic mechanism by which to reconcile vaccination as a disease-control tool alongside export certification through specific diagnostic testing modalities [199]. Implementation of such a strategy would support post-vaccination surveillance that documents absence of infection rather than simply documenting immune responses to vaccines. International organizations and national veterinary authorities increasingly emphasize DIVA-aware surveillance when vaccination is used as a complementary measure to stamping out disease and biosecurity prevention alone.

Operationalizing DIVA within vaccination development and implementation strategies requires pairing a marker or subunit of a vaccine with companion diagnostic tests that target antigens absent from the vaccine and integrating these tools into post-vaccination surveillance plans. While DIVA vaccination permits more flexible outbreak responses, such as suppressing transmission in high-risk zones or protecting valuable flocks, its success is dependent on diagnostic test sensitivity and specificity, the vaccine’s antigenic match to circulating strains, and surveillance intensity of the region [67,197]. Limits of current DIVA approaches, such as antigenic drift, variability in individual immune responses, and assay performance within field conditions, mean that vaccination programs against avian influenza in the poultry industry must be designed with clear DIVA protocols, robust validation, and transparent reporting to trade partners.

### 6.2. Limitations of Current DIVA-Compatible Diagnostics

The concept of a DIVA diagnostic test rests on the exploitation of immunological differences between vaccine-induced and infection-induced host responses. Ideally, a DIVA diagnostic would target antigens present in the virus during replication but absent or minimally present in the vaccine preparation or platform. However, implementation of DIVA in the field has proven difficult, in part because natural infection does not always elicit consistent antibody responses against marker antigens and due to some vaccine formulations inadvertently including small quantities of the same antigens. For example, serological approaches based on detecting antibodies in the host to non-structural protein NS1 of avian influenza virus—which is expressed only in infected cells and not packaged into virions—was originally proposed as a robust basis for DIVA, as inactivated vaccines should not contain NS1 [197,200]. Despite this accepted “rule” noticed in tissue culture and in vitro studies, empirical studies have shown in many birds infected post-vaccination that a minority (often less than 40%) of the flock seroconverts against NS1 within 3 weeks post-infection [201]. This low and variable sensitivity within a vaccinated population undermines the hypothesized reliability of NS1-based DIVA tests. This would be exceptionally difficult when applied to large flocks or in surveillance contexts where missing a few infected birds may have serious epidemiological consequences.

In addition to poor sensitivity, variability in individual immune responses, subclinical infections, and low-level virus replication can complicate the interpretation of serology-based DIVA platforms. In many vaccinated birds, virus exposure may result in limited replication or rapid clearance and thus produce a weak or transient antibody response to marker proteins [197,202]. Additionally, cellular immunity is often more efficient and critical at removing viral infections and, as such, serological testing may not encompass the entire picture of the host immune response when combating vaccine-induced responses and those from unregulated environmental exposure. Antigenic variability and differences in immune kinetics across different viral strains or host species may further reduce test sensitivity or lead to inconsistent performances across outbreaks [203]. Therefore, there is often no “gold standard” for defining “true infection” in vaccinated flocks, as the unknown true infection status confounds the validation of DIVA assays, and published estimates of sensitivity or specificity of probable DIVA platforms should be interpreted with caution.

Concrete examples illustrate these limitations. A study by Talazadeh et al. in 2014 [204] evaluating a commercial ELISA kit for general AIV antibodies found that sera from both vaccinated and infected chickens at either 3 weeks after vaccination or challenge tested positive. The difference in mean optical density between the groups in this study was also very minimal. The authors of that study concluded that this commercial assay could not discriminate vaccinated-only from infected birds [204]. Another investigation by Avellaneda et al. in 2010 using a recombinant NS1 protein ELISA in birds vaccinated with an inactivated AIV oil emulsion vaccine found that only a small proportion of challenged birds seroconverted for NS1, thus limiting the utility of NS1-based DIVA under these conditions [201].

Alternative DIVA strategies relying on subtype- or antigen-specific antibodies have also encountered challenges. For example, assays that target NA antibodies using a vaccine formulated using heterologous NA subtypes can theoretically allow for discrimination, but their field reliability is still a concern. Newer assays, such as NI enzyme-linked lectin assays (NI-ELLA), show promise, with a recent study reporting high specificity and good sensitivity for detecting infection in vaccinated and challenged birds, outperforming some NP-based ELISAs and RT-qPCR in experimental context [205,206]. However, broader validation across strains, species, and real-world field conditions remains a necessity before implementation of the assay and widespread reliability for surveillance or trade certification purposes.

In summary, while DIVA remains conceptually attractive and methodologically feasible, current serological diagnostics suffer from significant limitations, such as variable sensitivity, inconsistent immune responses, reliability of the antibody as a correlate of immune protection, potential antigen carry-over in vaccines, and the lack of a gold standard for assay validation. These challenges underscore the growing need for more robust, sensitive, and field-adaptable DIVA-compatible diagnostics if vaccination is to be used more broadly as a primary control strategy without compromising surveillance and trade efforts.

### 6.3. Biosensor Strategies for DIVA Compatibility

Incorporating biosensor technologies into a DIVA framework offers a promising avenue to help overcome many inherent limitations to conventional serology. In principle, two complimentary biosensor-based strategies may support DIVA platforms: direct detection of viral antigens (or virions) to signify active or recent infection and detection of host antibodies raised against antigen(s) not included in the vaccine formulation (“marker antigens”) or detection of vaccine-specific signatures in the special cases of recombinant or subunit vaccines. Each approach presents distinct advantages and disadvantages, but recent advances suggest that biosensor platforms may enable improved sensitivity and rapid, field-deployable DIVA diagnostic options.

#### 6.3.1. Viral Antigen/Virion-Based Biosensor Detection

Biosensors targeting viral antigens allow for the direct identification of viral particles or proteins within biological samples (e.g., swabs, environmental samples) independent of host serological status. Recent studies have demonstrated the feasibility of this approach for AIV. For example, an electrochemical-impedance-based immunosensor utilizing broadly reactive anti-M1 (matrix protein 1) antibodies attached to gold electrodes achieved detection of various IAVs with sensitivity approaching that of molecular methods within a much shorter resulting timeframe [71]. Another study employed novel capacitive biosensors composed of graphene oxide and Prussian blue modified electrodes for the detection of airborne H5N1 AIV in aerosol samples with a LOD of ~56 viral RNA copies/mL and a detection time of under five minutes [72]. Additionally, “cell-mimetic” nanoparticle-based biosensors have been developed for AIV detection; these nanoparticles mimic the host cell membrane and exploit the virus’ fusion machinery (HA to sialic acid moieties) to trigger a detectable signal (e.g., FRET-based) upon binding or internalization. This approach effectively detects a broad range of both LPAI and HPAI viruses [75].

Antigen/virion-based biosensor approaches hold value for DIVA implementation; they signal the presence of a virus and not just prior exposure and, as such, can unambiguously identify field infections even in vaccinated flocks. This is relevant for subclinical or early infections that could be detrimental in large herd and flock scenarios or in animals that may not mount robust antibody responses. For surveillance and trade certification contexts, antigen detection biosensors could provide a powerful complement or even an alternative to traditional serological diagnostic testing methods.

However, antigen detection methods have their own limitations. They usually need a sufficient viral load, whether from shedding or the environment. They may also miss infections that are latent or below the detection threshold. Sample quality, biosensor stability, and possible cross-reactivity continue to challenge antigen-based DIVA biosensors. To improve detection sensitivity, these methods might need to work alongside strong sampling protocols and possibly involve repeated scheduled testing.

#### 6.3.2. Host–Antibody (Serology-Based) Biosensors and Recombinant/Marker–Vaccine Detection

An alternative or complementary biosensor within a DIVA strategy leverages the detection of host–antibody responses either against “marker” antigens (absent from the vaccine) or against antigens uniquely present in recombinant vaccines. In conventional DIVA strategies, marker-based serology aims to discriminate antibodies induced from natural infection (such as non-structural proteins like NS1 or proteins not included in the vaccine design) from antibodies induced through vaccination. When translating this into a biosensor format, options such as immunosensors, aptasensors, or nanomaterial-enhanced serological assays offer several potential advantages. These advantages range from enhanced sensitivity and lower limits of detection compared to standard ELISAs to reduced assay time and processing and adaptability to POC or even field settings [202,207].

Recent reviews of biosensors have shown that nanomaterial-based immunosensors, such as gold nanoparticles, carbon nanomaterials, and quantum dots for antigen or antibody detection, have achieved very low detection limits, rapid turnaround times, and even portability. Electrochemical (EC) sensors functionalized with antibodies, aptamers, or other probes have been developed and increasingly used for virus detection. It is conceivable that these sensors could be tailored to detect host antibodies against marker antigens [208].

When considering subunit vaccine platforms as an option, biosensor-based serology could be designed to detect vaccine-specific antigen(s), such as a tagged HA subunit or a non-viral carrier antigen, or, conversely, to detect non-vaccine viral antigens, such as nonstructural proteins that would only appear upon natural infection. Such a strategy would mirror classical DIVA but with the benefits of biosensor speed, sensitivity, and lower infrastructure requirements. Combining antibody-based biosensors with multiplexing would also allow for more robust discrimination between vaccinated-only, infection-only, and vaccinated and infected animals, which would be incredibly valuable for complex field conditions or mixed-flock populations, especially in the face of timely outbreak responses.

#### 6.3.3. Feasibility and Practical Considerations

While technological potential is evident, there have been few published studies that have explicitly implemented biosensor-based DIVA strategies. Most published biosensor work for AIV remains focused on antigen or virus detection in naive or experimentally infected birds or general immunosensing. Realizing biosensor-based DIVA will therefore require strategic pairing of both vaccine design and biosensor assay development. Ideally, a recombinant/vectored or subunit vaccine should be used to exclude certain viral antigens from immune stimulus, and companion biosensor assays should be developed to detect either antibodies against antigens absent from the vaccine (infection markers) or antigens/epitopes unique to the vaccine (vaccine markers). Recombinant viral vectored vaccines for AIV are already described as DIVA-compatible and therefore provide a promising framework for biosensor integration and implementation [209]. Table 8 summarizes some promising biosensor platforms and how they could, theoretically, be applied to DIVA strategies [210,211,212,213,214,215,216,217].

The convergence of advanced biosensor technologies with rational vaccine design holds real promise to deliver field-deployable, rapid, and reliable DIVA diagnostics. The realization of this potential will necessitate coordinated development, encompassing both the pairing of vaccines and companion biosensors from the outset and rigorous validation in vaccinated and challenged flocks under field conditions.

## 7. Field Deployment and Point-of-Care Considerations

Point-of-care testing (POCT) has transformed the diagnostic process through the simplification of diagnostic approaches, reducing turnaround times and increasing accessibility, especially in limited resource environments [218]. Although centralized testing is robust and comprehensive, it suffers from logistical barriers stemming from sample transport and the need for specialized personnel and infrastructure [218]. POCT platforms are designed to be used at or near the site of patient care, facilitating delivery of accurate, real-time results, enabling clinicians to make immediate, data-driven decisions [219]. These benefits have not only addressed the limitations of conventional laboratory diagnostics but are especially critical in time-sensitive scenarios where early identification is essential for disease containment [218,219]. Beyond disease containment, POCT has been used in routine clinical management of viral infections and ongoing patient monitoring, displaying the added importance of integrating POCT into public health systems [218].

In limited resource environments, the portability of POCT platforms is especially advantageous. Device miniaturization has further enhanced the portability of POCT platforms and made it feasible for diagnostics to be performed in the absence of traditional lab infrastructure [218]. Furthermore, these tools align with One Health principles, enabling coordinated monitoring across human, animal, and environmental health systems [219].

### 7.1. Requirements for Field-Ready Diagnostics

Biosensor-based POC technologies hinge on several key attributes: portability, cost effectiveness, sensitivity, and ease of use. The innovative design of biosensors incorporates biorecognition elements with signal transduction systems to detect pathogens rapidly and with high specificity [218]. The compact and portable design of biosensors allows them to be used in decentralized settings, including schools, airports, and remote clinics [218]. This mobility enables real-time detection and intervention, which is important during outbreaks where reliable and fast diagnosis can be instrumental in halting disease spread.

Miniaturization has been instrumental in the advancement and shaping of field-ready diagnostic devices. For example, microfluidic-based POC technologies have consolidated the complexities of laboratory settings into small, contained units that are capable of operating in low-resource environments [220]. These devices deliver diagnostics at a fraction of the traditional cost, which promotes the accessibility of healthcare options [220]. However, there are still challenges that need to be addressed, such as the complexities of detection in varying environmental conditions and quality assurance.

Nanomaterials, such as graphene oxide, have improved the field performance of biosensors through the enhancement of sensitivity, durability, and signal amplification [220]. The continued evolution of biosensor technology will no doubt be important for the transformation of global health surveillance and diagnostics, especially in underserved regions.

### 7.2. Sample Matrices and Pre-Analytical Processing

Clinical specimens like tracheal and cloacal swabs, feathers, and serum are most often used in AI diagnostics. In conventional diagnostics, specimens are typically processed in Brain Heart Infusion (BHI) broth containing antibiotics and then transported on dry ice or by freezing to centralized labs for downstream analysis [221]. Specimen type and collection, processing, and preservation are some variables that can significantly impact diagnostic outcomes, especially in resource-limited environments.

Whilst RT-PCR is considered the gold standard in laboratory-based diagnostics for HPAI, as previously mentioned, this technique is limited by infrastructural demands and skilled personnel [222,223,224]. Thus, there is a need for robust, low-cost tests that can perform consistently across these sample matrices without requiring advanced equipment. There have been recent efforts in the development of paper-based immunoassays that use gold nanoparticles and HA-specific antibodies [222]. These platforms are designed to deal with the complexities of specimen types and deliver results in a sensitive and specific manner [222]. Feathers are considered a particularly effective sample type for AIV detection in asymptomatic birds [225]. In comparison to oral and cloacal swabs, feathers may harbor higher viral loads for an extended period due to the non-immunogenic nature of feather epidermal tissue [225]. Given their ease of collection, feathers can potentially be an ideal matrix for early detection and field-based diagnostics.

Despite major advances in analyte detection, there remains a gap in the development of cost-effective, low-tech solutions for specimen collection and handling that are compatible with POC platforms [226]. Methods for plasma separation and nucleic acid extraction are typically dependent on expensive instruments, which introduces a limitation to field applicability. Whilst there have also been promising separation techniques, these advancements have not been fully scalable or led to commercially available tools [226].

### 7.3. Integration with Digital and Mobile Platforms

The integration of biosensing technologies with smartphones and digital platforms is a promising way forward for POC diagnostics, especially in regard to adaptability for mobile healthcare applications. Smartphones and digital platforms use optical and electrochemical properties to detect signals from samples, enabling users to receive and interpret results in real time [227]. For smartphones in particular, the cameras can analyze changes like colorimetric and fluorescent signals, whilst electrochemical sensing involves detecting electrical responses from biochemical reactions using the phones’ USB ports or microphones and other audio interfaces [227].

A notable example of this integration is the Portronicx system, which is a smartphone-compatible electrochemical biosensor for dengue detection. This system works by using a wireless potentiostat and an Android app, which can detect viral antigen within 20 s [227].

Beyond detection, these mobile systems are also transforming diagnostics with cloud connectivity and remote data access. Real-time data sharing was invaluable during the COVID-19 pandemic by easing the load on centralized laboratories and supporting disease tracking on a broader scale [227]. Thus, the integration of biosensing technologies with mobile and smartphone platforms not only increases accessibility to timely healthcare but can be especially impactful for remote or resource-limited environments [227].

Smartphones have evolved into powerful biosensing platforms due to advancements in hardware and artificial intelligence. Innovations such as nanomaterial-based signal amplification, miniaturized microfluidic chips, and passive sensing technologies like near field communication (NFC) and radio frequency identification (RFID) have significantly improved sensitivity and portability. Overall, the convergence of biosensors with mobile and artificial intelligence technologies may usher in a new era of decentralized, intelligent, and globally accessible diagnostics [228].

### 7.4. Pilot Studies and Deployment Examples

Field-deployed diagnostics has become a cornerstone in outbreak response, especially in settings where centralized laboratories are not as accessible. During the Ebola crisis, the European Mobile Lab was an initiative that allowed for advanced diagnostic operations, including PCR, ELISA, and parasite microscopy, in the epicenter of the outbreak [219]. The lack of dependency on infrastructure bypassed the need for sample transport, allowing for timely diagnostic services to be conducted [219]. The mobile suitcase laboratory, initially designed for AI detection, was also vital during the Ebola outbreak in Guinea. This compact setup enabled nucleic acid extraction and amplification using isothermal and nanopore techniques in a low-resource environment [219], highlighting the importance of the deployment of field diagnostic tools in managing outbreaks.

The COVID-19 pandemic further underscored the relevance of decentralized testing; the deployment of POC molecular tests, such as Cepheid’s Xpert Xpress and Abbott’s ID NOW, was critical in accelerating the identification of individuals with high viral loads [219]. This enabled intervention and quarantine of infected individuals to be swift and guided the durations of these quarantine periods.

However, the pandemic also peeled back shortcomings in the rush to approve tests, which led to varying performance of tests and highlighted the lack of standardization of protocols for emerging pathogens [219]. Tracking viral load through repeated testing can inform discharge timelines and optimize hospital resource allocation [218]. Although rapid antigen tests offer speed, they often trade sensitivity as a result, especially in cases of early or low-load infections. Thus, the integration of molecular testing remains crucial to ensure diagnostic reliability, especially in high-risk or outbreak-prone settings [218].

Another important lesson of these deployment examples is the value of proactive surveillance and biosecurity. The zoonotic nature of strains like H5N1 and H7N9 necessitated strong animal–human interface monitoring. Measures such as culling, disinfection, and stringent hygiene in poultry operations have shown to be effective in controlling spread [229]. Additionally, the potential for antigenic shift and drift in influenza viruses has driven the creation of global surveillance frameworks under WHO guidance, essential for vaccine forecasting and preparedness. The current panzootic H5N1 wave has reinforced the need for coordinated surveillance, robust vaccine development pipelines, and continued investment in outbreak-ready diagnostic infrastructures [229].

## 8. One Health Implications and Surveillance Integration

The One Health approach provides a vital framework for understanding and managing AI surveillance across human, animal, and environmental health sectors. In the Asia–Pacific region, most members conduct some form of passive surveillance for HPAI, primarily through reporting and investigation of poultry and wild bird mortality events [230]. However, reporting delays often occur at the community level as a result of economic concerns and the capacity for active routine surveillance, such as sampling in live bird markets, commercial flocks, and wild bird populations [230]. Diagnostic capabilities for AI subtyping also vary widely between countries, further exacerbating these gaps. Although reports to the WOAH are generally timely, delays can occur. Furthermore, human surveillance systems, including severe respiratory infection monitoring, are in place but have not yet been fully integrated with animal health data. Environmental surveillance, despite its potential as an early warning system, remains underutilized [230,231].

Collaboration between organizations, such as WOAH and the Food and Agriculture Organization of the United Nations (FAO), models a practical application of the One Health principles by fostering data sharing between animal and health sectors [230]. These organizations make critical contributions to virological and epidemiological data, particularly for zoonotic AI subtypes, such as H5, H7, and H9, in WHO biannual vaccine composition meetings. This contributes to vaccine development and strengthens pandemic preparedness [230].

The integration of genomic surveillance, ecological studies, and modeling within a One Health context enhances early detection and cost effectiveness for avian and swine influenza surveillance. This has demonstrated a favorable cost to benefit ratio, reduces human disease burden, and offers substantial public health and economic benefits [231]. Future strategies should not only aim to address the health of people and domestic animals but also prioritize the sustainability of ecosystems. In the case of HPAI in wildlife, where response options are limited, prevention is paramount [232]. Reductions in viral emergence on poultry farms and preventing the spread into wild bird populations, particularly in shared habitats like wetlands, must be a priority [232]. The international community should also provide financial and technical support to low- and middle-income countries (LMICs) to expand AI surveillance in wildlife, thereby enhancing global pandemic resilience.

### 8.1. Zoonotic Risk and Public Health Relevance

AIVs, particularly highly pathogenic strains like H5N1, pose a zoonotic threat and have various public health consequences. AIVs are known for their genetic variation and ability to infect different species. They have evolved into many subtypes based on variations in their HA and NA surface proteins [233]. Since it was first detected in poultry in the mid-20th century and its first known transmission to humans in the late 1990s, H5N1 has been responsible for many human cases and fatalities worldwide [233].

Migratory wild birds play a key role in the global spread of these AIVs. These birds are often asymptomatic carriers capable of introducing viruses into new regions. A notable example is the 2.3.4.4b clade of H5N1, which spread from Europe to North America via migratory flyways and contributed to extensive outbreaks in both domestic and wild bird populations across the Americas [234]. Contrastingly, strains like H9N2 are endemic to regions like China and Egypt. H9N2 typically circulates within poultry and live bird markets (LBMs), with the potential to spread to humans [234]. These markets, where various species of birds are housed and sold in close quarters, are known hotspots for zoonotic transmission [234,235,236]. Environmental contamination in LBMs has been linked to risk factors including the type and number of birds sold, hygiene practices, and the duration of market operations [235].

Modeling studies suggest that short-lived market stays by infected birds can sustain virus circulation once favorable transmission conditions are met [236]. Practices like routine cleaning, rest days, and structural improvements may alleviate the risk of transmission; however, the effectiveness of these practices depends on proper implementation and consistent management. These findings highlight the critical need to prioritize surveillance and control efforts at the human–animal interface, especially in high-risk environments like LBMs, to curtail the emergence of novel AIV strains with pandemic potential.

### 8.2. Biosensors in One Health Surveillance Systems

Biosensors are emerging as a critical component of One Health surveillance systems, particularly in the context of zoonotic diseases like HPAI H5N1. Although human infections remain rare, the virus’ capacity for cross-species contamination has underscored the importance of early detection strategies at the human–animal–environment interface [233]. Surveillance challenges are impacted by the scale and speed of recent outbreaks, which have had far-reaching public health, ecological, and economic consequences. Traditional diagnostic methods, such as RT-PCR and ELISA, although accurate, are often time-consuming and rely on laboratory infrastructure, limiting their in-field applicability [32].

These limitations can potentially be addressed by biosensors, as these devices offer a portable, rapid, and increasingly sensitive means of detecting AIVs across various hosts and environmental samples. These devices integrate biorecognition elements, such as antibodies, aptamers, or glycans, with transducers to convert viral detection into visual, fluorescent, or electrochemical signals [32]. Recent innovations highlighted by Chen et al. have enhanced biosensor performance by incorporating isothermal amplification techniques like Reverse Transcription LAMP (RT-LAMP) and Rolling Circle Amplification (RCA), as well as plasmonic heating and CRISPR-based systems [237].

Some platforms eliminate the need for nucleic acid amplification altogether, allowing for real-time detection of unamplified RNA with high specificity and low detection limits. These advancements not only improve the outbreak response in poultry populations but also support continuous environmental monitoring, such as in wastewater or wildlife reservoirs, expanding surveillance capabilities across interconnected ecosystems. The improvement in scalability and field deployability of biosensors has immense promise for strengthening early warning systems and guiding interventions at the convergence of animal, human, and environmental interfaces

### 8.3. Global Health Equity and Low-Resource Settings

Access to accurate and timely diagnostics remains a critical challenge in many low-resource settings, where healthcare and infrastructure are often limited. Biosensors offer great potential for overcoming this challenge by enabling decentralized POCT that can be performed outside of traditional laboratories [220]. The ability to conduct rapid diagnostics at the site of care is particularly vital in remote or underserved communities, where delays in sample transport and processing can impede timely treatment. Technologies such as paper-based assays, portable microfluidic devices, and smartphone-integrated platforms provide low-cost, easy-to-use alternatives that do not rely heavily on external reagents or power sources, addressing many of the logistical barriers faced in these environments [224,238].

However, the effective deployment of biosensors in low-resource settings requires overcoming a complex mix of technical, economic, and operational hurdles. Criteria for biosensors include high reproducibility, robustness, and capacity for long-term storage without the need for refrigeration whilst simultaneously minimizing environmental waste [238]. Beyond technical specifications, the cost of both development and implementation is a major limiting factor. Most innovations in biosensors have been determined by markets in developed countries, often not considering cost-effectiveness or affordability in LMICs [238]. Furthermore, regulatory approval pathways for diagnostics remain weak or inconsistent in many LMICs, increasing the risk of unregulated or substandard products entering the market [224,238].

Another challenge lies in the level of expertise of the healthcare workforce. Sample collection, storage, and preparation often require trained personnel and specialized equipment, which may be scarce in resource-limited settings [224]. Additionally, interpreting diagnostic results and implementing them into clinical decision making require a workforce equipped with adequate training and support. Without proper investments in training, quality management, and supply chain infrastructure, biosensor deployment risks being undermined by misdiagnosis, device wastage, or misuse. Concerted efforts involving governments, healthcare providers, funders, and manufacturers are therefore essential to build the necessary competencies and ensure biosensors translate into meaningful health outcomes [220,239].

To maximize their impact, biosensor development for global health equity should prioritize affordability, simplicity, and adaptability. Embracing open-source hardware designs and inexpensive materials can reduce costs and facilitate local manufacturing, improving supply chain resilience [238]. Integration with mobile technologies offers opportunities for real-time data transmission and remote monitoring, enhancing surveillance at the intersection of human, animal, and environmental health [226]. Ultimately, successful deployment will depend not only on technological innovation but also on strategic policy frameworks, sustainable financing, and inclusive partnerships that address systemic barriers [226,238,239].

## 9. Challenges, Gaps, and Future Directions

Despite significant progress in the development of biosensor platforms for AI detection, substantial challenges remain that limit widespread adoption, field reliability, and regulatory acceptance. Addressing these gaps requires not only advances in engineering and validation but also coordinated efforts across disciplines and the integration of emerging technologies that can reshape the diagnostic landscape.

### 9.1. Technical Barriers and Standardization Needs

Reproducibility, cross-reactivity, and long-term stability limit reliable translation of biosensor prototypes into field-ready DIVA tools. While many biosensor studies in this field are promising, reports of excellent analytical sensitivity are based on buffered or spiked matrices and do not demonstrate equivalent performances in complex, real-world samples and matrices, such as avian swabs, environmental dust, or pooled specimens. This gap causes reproducibility concerns when moving from laboratory testing on the bench to field use. The absence of standardized sample-handling protocols, certified reference materials, and inter-laboratory data for biosensor platforms undermines the comparability of limits of detection, precision, and specificity across these studies.

Cross-reactivity with related viruses or subtypes along with environmental interferents is a hazard for DIVA biosensor development; AIV antigens and conserved internal proteins can share epitopes across different subtypes and other avian pathogens, while environmental matrices, such as dust and fecal material, can introduce nonspecific binding or signal quenching. Biosensors designed to rely on a single epitope for recognition, such as monoclonal antibodies or single aptamers, risk false positives in mixed species or multi-pathogen settings unless they are validated against broad cross-reactivity panels and field samples. Likewise, serology-based DIVA markers, such as NS1, are conceptually attractive, but, in practice, they have shown variable seroconversion after infection and, as such, compound diagnostic uncertainty unless sampling and assay timing considerations are rigorously defined [197,240].

Long-term stability and operational robustness determine whether a biosensor is “fit” for on-farm use. Biological recognition elements (antibodies, enzymes, aptamers), nanomaterial surfaces, and polymer matrices can degrade under heat, humidity, or mechanical stress, producing lot-to-lot variability or drift in signal over weeks to months. Devices intended for low-resource use or field deployment should thus undergo accelerated and real-time stability testing, such as temperature and humidity cycling, shelf-life studies, and repeated-use endurance tests, to be benchmarked against standards used for established diagnostics. Embedding stability evaluation, reference calibrants, and clear storage and handling instructions into early device development is essential to move from a promising prototype to a reliable biosensor that can be used as a field instrument [241].

**Figure 4:**
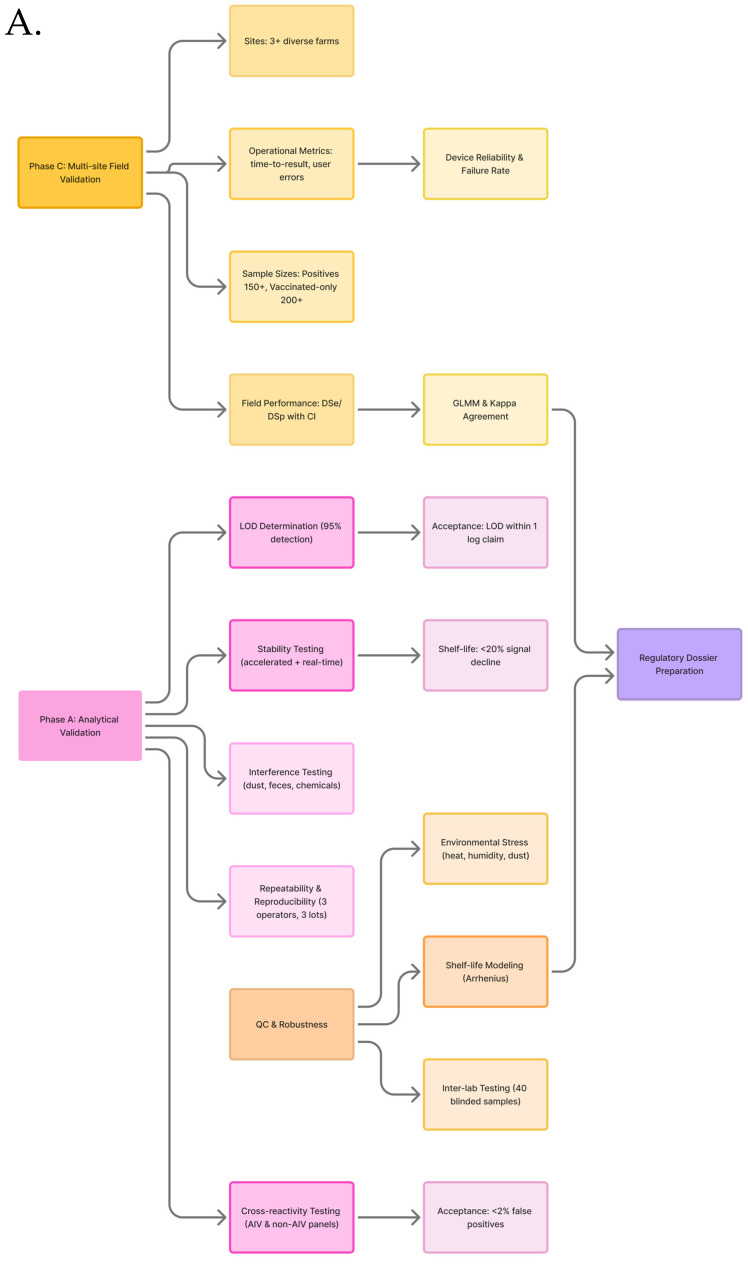

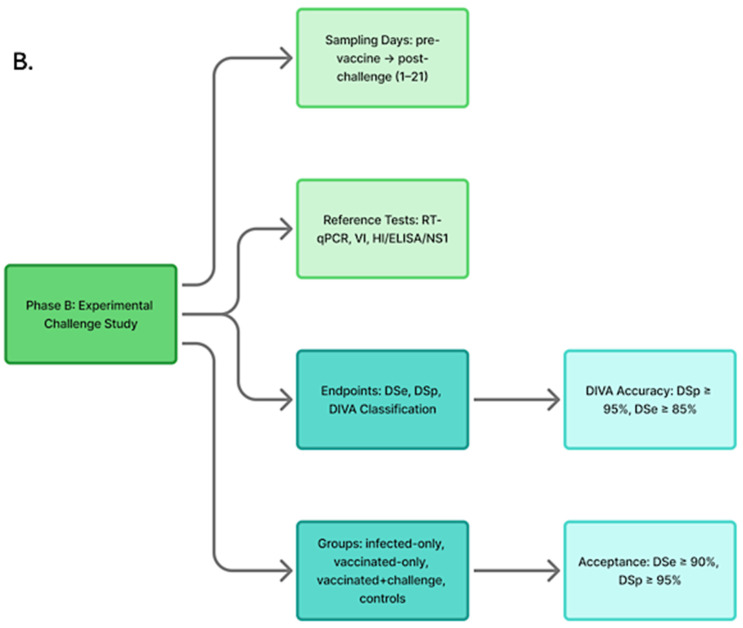
A proposed workflow for moving benchtop lab development of biosensors to a scalable in-the-field biosensor testing modality for HPAI. (**A**) Phase A, analytical validation, and phase C, multi-site field validation. While phases A and C are not sequential, their end results are both regulation dossier filing. (**B**) Phase B, experimental challenge study, which should take place prior to phase C.

Figure 4 examines a proposed workflow for moving a biosensor lab platform to field implementation.

### 9.2. Regulatory and Validation Challenges

Transitioning a biosensor device from prototype to commercial AIV diagnostic capacity requires a clearly documented validation pathway demonstrating analytical performance, clinical utility, manufacturing quality, and post-market oversight. International veterinary authorities, such as the WOAH, maintain validation frameworks and registration for diagnostic kits, and their Terrestrial Manuals defines “fit-for-purpose” validation criteria beyond single-laboratory LOD claims to include diagnostic sensitivity and specificity, intended use, and quality management. These criteria must be fulfilled by any biosensor, especially DIVA biosensors, to meet trade-relevant acceptance. Early engagement with regulatory authorities in designing validation studies to align with WOAH/OIE templates, such as multi-site field trials and submission dossiers, will accelerate regulatory review and acceptance [242,243].

Analytical performance claims are necessary but not sufficient, as regulators and end users require epidemiological and clinical validation to show that the device can reliably differentiate vaccinated-only, infected-only, and vaccinated and infected birds in the intended production settings, such as different species, ages, and vaccine types. Achieving this requires large prospective field studies or well-designed challenge models with representative sampling frames. Parallel to clinical studies, manufacturers must demonstrate manufacturing consistency (ISO 13485 quality systems) and diagnostic lab handling consistency (ISO 17025), lot-to-lot reproducibility, and robust supply chains for critical reagents to satisfy regulatory and purchaser requirements [244].

Practical adoption goes beyond just formal validation. It relies on several operational factors, including user training, device durability, cold-chain needs, data reporting and connectivity, and integration with surveillance workflows accepted by regulators and trading partners. Successful adoption paths in rapid diagnostics include three key elements: (a) a thorough analytical and field validation study, (b) clear specification of use cases, such as on-farm screening versus laboratory confirmation, and (c) strategies for linking data for reporting and confirmatory testing [241]. These elements are what biosensor developers should plan for from the prototype stage before engaging in field deployment and real-world application testing.

### 9.3. Research Priorities and Interdisciplinary Collaboration

Developing strong biosensors for AIV and DIVA applications involves many fields. Engineering teams create new transducers and recognition chemistries. Virologists and immunologists provide validated antigens, study marker dynamics, and design challenge models. Veterinarians and epidemiologists set up sampling plans and surveillance levels. Public health officials and trade stakeholders outline their needs for export certification and response actions. Successful translation, therefore, requires integrated consortia and codesigned validation studies that bring these individuals together early on in the device development lifecycle. PREDICT/One Health lessons have shown that coordinated, multisectoral collaborations substantially improve surveillance design and outbreak response—a template that biosensor development efforts and researchers should emulate [245].

Concrete research priorities include the creation and wide distribution of standard reference materials, including inactivated virus panels and characterized sera from known vaccination or infection histories. Furthermore, priorities should also include curated cross-reactivity panels covering endemic avian pathogens and other AIV subtypes and strains and multi-species longitudinal studies documenting antigen and antibody kinetics post-vaccination and post-infection across production pipelines. Comparative inter-platform studies, such as head-to-head comparisons of biosensors and molecular assays in the same field settings, will provide clarification of relative strengths and weaknesses, helping guide algorithmic integration. Investments in data standards and connectivity to enable pooled, anonymized field performance datasets will accelerate algorithm improvement and regulatory confidence [244].

Lastly, capacity building and stakeholder engagement are essential. Training veterinarians and field technicians in proper sampling for biosensors, creating open-access datasets to enable independent validation, and maintaining dialogue with trading partners and regulatory agencies about acceptable evidence thresholds for DIVA claims are all ways to assist with engagement and integration of all personnel. Such collaborative, cross-disciplinary frameworks increase the likelihood that biosensor innovations will be incorporated into surveillance programs and accepted for trade-sensitive decisions.

### 9.4. Emerging Technologies and Paradigms

Artificial intelligence and machine learning present powerful tools to improve the interpretation of biosensor outputs, especially when devices generate multiplexed or longitudinal data. Artificial intelligence models can denoise signals, deconvolve multiplex fluorescence or electrochemical fingerprints, and integrate temporal patterns to reduce false positives and flag likely true infections for confirmatory testing. Recent reviews highlight both the promise and challenges of embedding artificial intelligence within POC decision making and processing [246]. However, artificial-intelligence-assisted biosensor interpretation is accelerating multiplexed POC diagnostics and a logical fit for next-generation DIVA workflows and diagnostic platforms, like biosensors, that require synthesis of antigen, antibody, and contextual metadata.

Self-powered and wearable sensor technologies are maturing rapidly and could allow for enabling continuous environmental surveillance in poultry houses or wildlife roosts. Advances in ambient energy harvesting through solar, kinetic, or thermoelectric methods and hydrogel harvesters allow for low-power biosensor modules to operate autonomously for extended periods, transmitting alerts when viral signals cross action potential thresholds [247]. Wearable or attachable sampling devices for high-value birds or sentinel flocks, combined with on-board concentration and detection modules, could theoretically support longitudinal monitoring and early warning systems. However, practical constraints, such as cost per bird, animal welfare, and data management needs, require careful evaluation.

Lastly, the convergence of CRISPR-based diagnostics, cell-mimetic and synthetic receptor systems, microfluidic sample-to-answer cartridges, and networked data platforms points towards a new generation of integrated diagnostic solutions: low-cost, multiplex biosensors that combine antigen detection, genetic marker screening, and antibody profiling with artificial-intelligence-guided decision support and cloud reporting. Early lab-on-a-chip demonstrations for respiratory viruses, including IAV, show the technical feasibility of multiplexed, low-power, rapidly validated platforms [248]. Moving these into DIVA-specific, vaccinated-flock validation studies is a priority to demonstrate real-world implementation and potential impact.

## 10. Conclusions

### 10.1. Summary of Key Insights

Biosensor platforms represent a transformative opportunity for AI diagnostics by offering rapid, sensitive, and increasingly field-ready alternatives to conventional laboratory methods. Across diverse biorecognition strategies—including antibody-based systems, aptamers, molecularly imprinted polymers, CRISPR-based detection, and nanomaterial-enhanced platforms—biosensors demonstrate strong potential to overcome longstanding limitations related to turnaround time, portability, and cost. Their intrinsic modality allows for adaptation to evolving viral strains, integration of multiplex detection, and customization for surveillance, outbreak response, and DIVA frameworks. As this review highlights, these technologies bridge the gap between laboratory-grade analytical performance and POC utility, positioning biosensors as a novel tool in modern avian influenza detection ecosystems.

In addition to AI, biosensor technologies have already been applied successfully to a diverse array of viral pathogens of One Health importance, demonstrating the versatility and field-use potential of biosensor-based platforms. Rapid, portable biosensors have been developed for SARS-CoV-2, which causes COVID-19, including electrochemical, optical, and CRISPR-based devices that enable POC detection with reduced dependence on laboratory infrastructure compared to conventional quantitative PCR assays [249,250]. This highlights opportunities for decentralized surveillance in human and environmental contexts during pandemics. Also, biosensors targeting Zika virus have been explored with field-ready designs aimed at high sensitivity and specificity in resource-limited settings [251]. West Nile virus, another arbovirus with wildlife and human health implications, has been detected using fluorescence-based biosensors capable of lineage discrimination and application to clinical samples [252]. Broader reviews have also showcased electrochemical biosensing strategies for a multitude of viral diseases, such as influenza, Ebola, HIV, and others. This underscores the generalizable nature of biosensor approaches for viral diagnostics across sectors [253,254]. These examples illustrate that biosensor technologies are not only advancing for specific pathogens but increasingly being integrated into One Health frameworks spanning human, animal, and environmental surveillance needs.

### 10.2. Vision for Future AI Diagnostics

The future of avian influenza diagnostics and biosensors will be shaped by the integration of DIVA strategies with artificial intelligence, data connectivity, and real-time decision support. Machine learning offers the ability to refine signal interpretation and automate classification of infection status and viral subtype. When combined with DIVA-oriented strategies like antigen profiling and host antibody signatures, artificial-intelligence-assisted biosensors could achieve unprecedented precision in distinguishing infection from immunized animals.

While it is correct that the lack of DIVA capability has historically hindered US-based AI vaccination programs, several DIVA-compatible strategies have been explored. These include the use of heterologous NA vaccines, non-structural protein 1 (NS1)-based serology, and recombinant or vectored vaccines that omit selected viral antigens to enable serological differentiation between infected and vaccinated birds. However, these approaches remain largely dependent on centralized, conventional laboratory assays, such as ELISA or RT-qPCR, limiting their utility for rapid, large-scale field surveillance and complicating real-time outbreak decision making. Additionally, many existing DIVA assays lack sufficient sensitivity during early infection, are not easily multiplexed, or require paired testing strategies that increase logistical burden.

Biosensor technologies provide a clear opportunity to overcome these limitations by enabling rapid, decentralized, and multiplexed DIVA-compatible diagnostics. Emerging biosensor platforms can be designed to simultaneously detect antibodies against vaccine-excluded antigens (e.g., NS1), strain- or clade-specific hemagglutinin or neuraminidase epitopes, and molecular markers of active viral replication and transition of strains to HPAI variants. Electrochemical, optical, and CRISPR-based biosensors offer the potential for high specificity and subtype discrimination in portable formats. When integrated with DIVA-compliant vaccine designs, these biosensors could support real-time differentiation of infection versus vaccination at the POC, which would preserving surveillance integrity while enabling vaccination as a viable disease management strategy. Such advances would represent a critical step toward harmonizing vaccination, diagnostics, and trade requirements within avian influenza control programs.

Advances in mobile integration, wireless communication, and cloud-based analytics will extend the reach of these tools to make them more practical for remote or resource-limited environments. Field-ready systems incorporating microfluidics, self-powered materials, and rugged sensor components point toward highly autonomous platforms capable of delivering actionable results directly to veterinarians, wildlife managers, and public health authorities. In this future landscape, biosensors are not merely diagnostic devices but components of an interconnected surveillance network.

### 10.3. Final Remarks

As highly pathogenic avian influenza continues to influence global health, agriculture, and trade, the need for rapid, reliable diagnostic tools grows. Biosensors offer a promising way to expand surveillance, support faster outbreak responses, and strengthen vaccination programs. This is compounded especially when DIVA capabilities are built in. Their successful development, however, relies on close collaboration across different scientific and professional disciplines to solve complex diagnostic challenges.

Moving these technologies from promising prototypes to practical, deployable tools will also require sustained investment and early engagement with regulatory partners. These steps are essential for ensuring biosensors meet the standards needed for field use, international reporting, and disease-control decisions. Ultimately, advancing biosensor technology is not only about improving poultry health—it also plays a key role in reducing zoonotic risks and protecting the shared health of people, animals, and the ecosystems they depend on. Continued innovation in this space will be vital as we face an era of rapidly evolving infectious threats.

## Figures and Tables

**Table 2:** Comparison of AIV biosensor technologies.

Platform Class	Representative Sensor Example	Target	LOD (Standardized)	Assay Time	Applicable Sample Types	Key Limitations	References
Electrochemical (EIS)	Universal influenza A sensor (anti-M1)	M1 antigen/virion	NR (paper-specific)	<1 h	Biological matrices	Fouling; calibration; surface reproducibility	[71]
Capacitive electrochemical (GO/PB)	Aerosol AIV H5N1 capacitive sensor	RNA/virion	56 copies/mL	<5 min	Aerosols (sampler liquid), environmental	Sampler dependency; matrix effects	[72]
SPR (label-free)	Sandwich SPR aptamers for whole H5Nx	Whole virion	200 EID50/mL	~10–20 min (typical SPR run)	Buffer, clarified swab extract	Instrument cost; RI drift; needs stable temp	[73]
LSPR plasmonic (nanoparticle)	DNA 3-way junction HAuSN LSPR	H5N1 HA	1 pM (=1 fmol/mL)	~10–20 min	PBS; diluted chicken serum	May require sample dilution; optical stability	[74]
Cell-mimetic optical (fusion/FRET)	Cell-mimetic biosensor	Virion fusion signature	Not reported	NR	Biological environments	Often broad influenza, limited subtype	[75]
QCM aptasensor	Hydrogel-amplified QCM H5N1	Whole virion/HA	~0.0128 HAU/mL	~30–60 min	Processed swab eluate	Sensitive to viscosity/temp; flow control	[69]
MIP-QCM	Influenza MIP QCM subtype profiling	Virion/epitope	~10^2^–10^3^ PFU/mL	~30–60 min	Processed matrices	Template variability; validation needed	[68]

**Table 3:** Comparison table of Direct Antigen and Indirect Antibody detection tests used for highly pathogenic avian influenza (HPAI).

	Test Type	Principle	Time Required	Sensitivity	Specificity	Ease of Use	Cost	Portability	Sample Type	Common Use Case
Direct Antigen detection test	Rapid Antigen Test (Lateral Flow Assay—LFA)	Detects viral nucleoprotein (NP) via immunochromatography	10–30 min	Moderate (~60–80%)	High (~90–95%)	Very easy (minimal training)	Low	High (field-deployable)	Cloacal/oropharyngeal swabs, tissues	Field screening, quick diagnosis
	Enzyme-Linked Immunosorbent Assay (ELISA)	Antibody–antigen binding detected via enzyme reaction	2–4 h	High (~85–95%)	High (~95–99%)	Moderate (lab equipment needed)	Moderate-High	Low (lab-based)	Swabs, tissues, fluids	High-throughput lab testing
Indirect Antibody detection test	Rapid Antibody Test (Lateral Flow Assay—LFA)	Immunochromatographic detection of anti-HPAI antibodies	10–30 min	Moderate (~60–80%)	Moderate (~85–90%)	Very easy (field-friendly)	Low	High (portable)	Serum, whole blood	Field serology, quick screening
	Enzyme-Linked Immunosorbent Assay (ELISA)	Detects anti-HPAI antibodies via enzyme-linked secondary antibodies	2–4 h	High (~85–95%)	High (~90–98%)	Moderate (lab equipment needed)	Moderate	Low (lab-based)	Serum, plasma	High-throughput serosurveillance
	Hemagglutination Inhibition (HI) Test	Measures antibodies blocking viral hemagglutination (HA)	4–6 h	High (~80–95%)	High (~95–100%)	Moderate (requires standardized reagents)	Moderate	Low (lab-based)	Serum	Subtype-specific antibody detection
	Virus Neutralization Test (VNT)	Detects neutralizing antibodies through viral infectivity reduction	3–5 days	Very high (~95–100%)	Very high (~98–100%)	High skill required	High	Low (lab-based)	Serum	Gold standard for protective immunity

**Table 4:** Comparison table of commercial influenza A/B antigen detection tests.

Manufacturer	Device Name	Method	Detection Targets	Key Features
Quidel Corp. (San Diego, CA, USA)	QuickVue Influenza A + B Test	Immunoassay	Influenza A and B	Rapid visual read (10–15 min)
Quidel Corp. (San Diego, CA, USA)	Sofia Influenza A + B FIA	Fluorescent Immunoassay	Influenza A and B	Automated (Sofia Analyzer), higher sensitivity than QuickVue
Abbott Diagnostics Scarborough, Inc. (Abbott Park, IL, USA)	BinaxNOW Influenza A & B Card	Immunochromatographic	Influenza A and B	Rapid (15 min), no subtyping
Becton, Dickinson and Company (Sparks, MD, USA)	BD Veritor System (Flu A + B)	Digital Immunoassay	Influenza A and B	Digital reader reduces interpretation errors
CorDx, Inc. (San Diego, CA, USA)	Tyfast Flu A/B & COVID-19 Test	Multiplex Rapid Test	Flu A/B + COVID-19	Combined detection, 15 min result
Healgen Scientific, LLC. (Houston, TX, USA)	COVID-19/Flu A&B Combo Test	Lateral Flow	Flu A/B + COVID-19	Dual-target cassette (nasal/swab)
Sekisui Diagnostics, LLC. (San Diego, CA, USA)	OSOM Flu A&B Test	Immunochromatographic	Influenza A and B	High specificity
Princeton BioMeditech Corporation (Monmouth Junction, NJ, USA)	BioSign Flu A + B	Lateral Flow	Influenza A and B	Visual read, 10–15 min
Thermo Fisher Scientific (Waltham, MA, USA)	Xpect Flu A&B	Immunoassay	Influenza A and B	Moderate complexity, lab-based

**Table 5:** Comparison of aptamer-based methods for influenza virus detection.

Virus Strain	Target	Detection Method	Key Findings
H5Nx (avian influenza) [73]	Whole virus	Sandwich-type surface plasmon resonance (SPR) biosensor	- Detects intact virions without pretreatment - High specificity for H5Nx among avian strains
H3N2 [131,132]	Globular region of hemagglutinin (HA)	Aptamer-functionalized magnetic microparticles + colorimetric readout	- Rapid (<30 min) and equipment-free - Works in clinical samples (e.g., nasopharyngeal swabs)
H1N1 [129,133]	Inactivated virus particles	Electrochemical impedance sensor (EIS) + sandwich enzyme-linked oligonucleotide assay (ELONA)	- pg-level sensitivity (improved over ng-level ELONA) - Probe density optimization reduces steric hindrance
H1N1 variants [130]	Minin-hemagglutinin (stem region) or whole virus DNA aptamer	ssDNA aptamer-based electrochemical assay	- Differentiates H1N1 subtypes from its variants (seasonal H1N1, H3N2, and 2009 H1N1) - Cross-reactivity tested against H3N2, H5N1, etc.

**Table 6:** Comparison of CRISPR-based biosensing methods for HPAI detection.

Method	CRISPR System	Target Gene(s)	Limit of Detection (LOD)	Detection Time	Output Type	Strengths	Limitations
SHERLOCK (Specific High Sensitivity Enzymatic Reporter UnLOCKing) [153]	Cas13a	HA, NA, M gene	10–100 copies/μL	~1 h	Fluorescence/Lateral Flow	High sensitivity; RNA detection	Requires pre-amplification (e.g., RPA)
DETECTR (DNA Endonuclease Targeted CRISPR Trans Reporter) [154]	Cas12a	M gene	10 copies/μL	~30–60 min	Fluorescence/Lateral Flow	Rapid, visual detection	Mainly for DNA; needs amplification
CRISPR/Cas12a with RT-LAMP [155]	Cas12a	HA, M gene	1–10 copies/reaction	~45–60 min	Colorimetric or Lateral Flow	Isothermal; easy to adapt in field	Requires optimization of LAMP conditions
All-in-One Dual CRISPR-Cas12a (AIOD-CRISPR) [156]	Cas12a	HA gene	~1 copy/μL	~30–40 min	Fluorescence	One-pot, simple setup	Limited to lab conditions for now

**Table 7:** Comparison of nanomaterial-enhanced biorecognition of HPAI.

Nanomaterial	Target (Subtype)	Limit of Detection (LOD)	Linear Range	Detection Time	Detection Method/Platform
Gold nanoparticle [74]	H5N1	1 pM	1–10^4^ pM	10 min	UV–Vis Spectroscopy/Surface Plasmon Resonance (SPR)
Silver nanorod [163]	H7N9	31 × 10^−6^ pM (H7), 44 × 10^−6^ pM (N9)	10^−3^–10^14^ pM	7 h	Confocal Raman spectroscopy/Surface Enhanced Raman Spectroscopy (SERS)
AuAg^4–ATP^@AgNPs [164]	H7N9	1.8 × 10^2^ PFU	–	20 min	Naked eye + Raman/Surface Enhanced Raman Spectroscopy (SERS)
Nitrogen-doped carbon nanotube arrays decorated with AuNP [165]	H5N2	0.7 × 10^2^ PFU/mL	–	Few minutes	Raman spectroscopy/Surface Enhanced Raman Spectroscopy (SERS)
Multi-walled carbon nanotube- polydimethylsiloxane [166]	H5N1, H7N9, H9N2	55.7 pg/mL (H5N1); 99.6 pg/mL (H7N9); 54 pg/mL (H9N2)	10^2^–10^5^ pg/mL	20–30 min	Differential Pulse Voltammetry/Electrochemical
Boron-doped diamond [167]	H5N3, H7N1, H9N2	0.13 PFU (H5N3); 0.38 PFU (H7N1); 6.70 PFU (H9N2)	3–400 PFU	–	Electrochemical Impedance Spectroscopy/Electrochemical
Magnetic nanospheres + AuNPs [168]	H7N9	0.03 pg/mL	0.2–2 × 10^5^ pg/mL	–	Single-nanoparticle collision electrochemistry (SNCE)/Electrochemical
Enzyme-encapsulated liposome [169]	H5N1	40 pg/mL	100–4000 pg/mL	–	Naked eye + UV–Vis/Colorimetric
Silica nanoparticle [170]	H7N2, H7N9	0.08 pg/mL	10^2^–10^4^ pg/mL	15 min	Naked eye, camera + ImageJ software (1.53 t)/Luminescent
Porous silica nanoparticle [171]	H9N2, H5N9	0.7 × 10^3^ PFU/mL (H9N2); 0.7 × 10^4^ PFU/mL (H5N9)	–	–	Optical/Electronic Instrument/Luminescent
Magnetic imprinted polymer + Zn_2_GeO_4_:Mn^2+^ [172]	H5N1	0.013 × 10^5^ PFU/mL	(0.013–1.28) × 10^5^ PFU/mL	–	MIP-Aptasensor/Luminescent

**Table 8:** Promising biosensor platforms for potential DIVA strategies with their strengths and weaknesses.

Biosensor Platform	Biorecognition Strategy	How the Platform Enables DIVA	Key Strengths	Primary Limitations
ACEK-Enhanced Impedimetric Sensor [210]	Antigen detection (HA/NS1) with AC electrokinetic concentration	Detects higher NS1/HA levels during natural infection compared to vaccinated birds; antigen presence directly indicates active viral replication	Very high sensitivity due to analyte concentration Rapid (<10–15 min) Field-deployable and low-power	Viral antigen levels decline quickly Antigen drift reduces binding Less useful in later infection stages
Aptamer-Based Fluorescent Sensor (NS1 or HA) [211]	Synthetic single-stranded DNA aptamers with high target affinity	NS1 is expressed only during viral replication, not after inactivated vaccination; aptamers can differentiate HA variants	High specificity and chemical stability Low-cost synthesis, batch reproducibility Good adaptability to portable readers Performance sensitive to viral mutations	Requires a fluorescence detection module
Molecularly Imprinted Polymer (MIP) Electrochemical NS1 Sensor [212]	Synthetic polymer with tailored NS1-shaped binding cavities	Detects NS1 as infection-only marker absent from most conventional vaccines	Extremely rugged and stable No cold-chain; low-cost Excellent for field use	Lower binding affinity vs. antibodies/aptamers Template leakage possible Difficult to imprint large proteins accurately
CRISPR-Cas12 SHERLOCK Viral RNA Assay [213]	Cas12-mediated RNA recognition with collateral reporter cleavage	Targets internal gene regions that are not included in recombinant marker vaccines, allowing for molecular discrimination	Ultra-sensitive (<10 RNA copies) High sequence specificity Compatible with lateral-flow DIVA	Often requires nucleic acid extraction Regulatory uncertainty for CRISPR POCT Cold-chain for certain reagents
CRISPR-Cas13 DETECTR for Vaccine Marker Deletions [214]	Cas13 collateral cleavage triggered by engineered deletion markers	Designed for recombinant DIVA vaccines lacking specific genomic segments; detects presence of deletion sites	Highly precise for recombinant vaccine programs Rapid (<30 min) Strong potential for on-site genotyping	Only functional where marker vaccines exist Requires stable genetic engineering Higher development cost
Gold Nanoparticle (AuNP) NS1-Antibody Lateral Flow Assay [215]	AuNP–NS1 conjugates detect infection-derived anti-NS1 antibodies	Infected birds mount NS1-specific antibodies; vaccinated birds (inactivated) typically do not	Very low cost Rapid, easy to use Scalable to flock-level screening	Needs seroconversion (7–10 days) Antibody responses vary across birds Potential cross-reactivity
Carbon-Nanomaterial Electrochemical Immunoelectrode [216]	CNT-enhanced multi-analyte antibody profiling	Profiles differential antibody patterns (e.g., anti-HA vs. anti-marker antibodies) to distinguish vaccination from infection	High electrochemical sensitivity Quantitative and multiplexable Useful for complex vaccination programs	Humoral variability complicates interpretation Risk of cross-reactivity among subtypes
Quantum Dot (QD) Multiplex Immunoassay [217]	Fluorescent QD-tagged viral antigens and marker proteins	Simultaneously distinguishes infection antigens (HA/NA) from recombinant marker proteins via unique spectral signatures	Highly multiplexed Very strong signal amplification Low cross-reactivity	Requires fluorescence optics More complex and costly to manufacture

## Data Availability

No new data were created or analyzed in this study.
